# *MiR221/222* in the conditioned medium of adipose-derived stem cells attenuates particulate matter and high-fat diet-induced cardiac apoptosis

**DOI:** 10.1186/s13287-025-04381-8

**Published:** 2025-06-03

**Authors:** Ya-Chun Chen, Chi-Ming Pu, Shu-Rung Lin, Shu-Wha Lin, I-Shing Yu, Ho-Jun Shih, Chiang-Wen Lee, Ming-Hsueh Lee, Yen-Ting Kuo, Yuh-Lien Chen

**Affiliations:** 1 Department of Anatomy and Cell Biology, College of Medicine, National Taiwan University, No. 1, Sec 1, Ren‐Ai Road, Taipei, 10051 Taiwan, ROC; 2 Division of Plastic Surgery, Department of Surgery, Cathay General Hospital, Taipei, Taiwan, ROC; 3 School of Medicine, College of Life Science and Medicine, National Tsing Hua University, Hsinchu City, Taiwan, ROC; 4 Department of Bioscience Technology, College of Science, Chung Yuan Christian University, Taoyuan, Taiwan, ROC; 5 Center for Nanotechnology, Chung Yuan Christian University, Taoyuan, Taiwan, ROC; 6 Department of Clinical Laboratory Sciences and Medical Biotechnology, College of Medicine, National Taiwan University, Taipei, Taiwan, ROC; 7 Laboratory Animal Center, College of Medicine, National Taiwan University, Taipei, Taiwan, ROC; 8 Department of Respiratory Care and Chronic Diseases and Health Promotion Research Center, Chang Gung University of Science and Technology, Chiayi, Taiwan, ROC; 9 Division of Neurosurgery, Department of Surgery, Chang Gung Memorial Hospital, Chiayi, Taiwan, ROC; 10 Department of Respiratory Care, Chang Gung University of Science and Technology, Chiayi, Taiwan, ROC; 11 Department of Emergency Medicine, Chang Gung Memorial Hospital, No. 8, Sec. West, Jiapu Rd., Puzi City, Chiayi, 61363 Taiwan, ROC

**Keywords:** Particulate matter (PM), Palmitic acid (PA), High-fat diet (HFD), Conditioned medium from cultured adipose-derived stem cell (ADSC-CM), *miR221/222*, Reactive oxygen species (ROS), Mitochondrial fission

## Abstract

**Background:**

Air pollution and obesity are crucial risk factors for cardiovascular disease (CVD), with epidemiological evidence indicating that air pollution exacerbates obesity-induced cardiac damage. Treatment with adipose-derived stem cells (ADSCs) attenuates cardiac damage by releasing paracrine factors. However, the effects of ADSCs on air pollution- and obesity-induced cardiomyocyte apoptosis and the related mechanisms are still unclear.

**Methods:**

Palmitic acid (PA) and a high-fat diet (HFD) were used to cause obesity, and particulate matter (PM) was used to simulate air pollution in the study. We studied the impact of conditioned medium from adipose-derived stem cells (ADSC-CM) on the apoptosis of PA + PM-treated H9c2 cells and HFD + PM-treated mouse cardiomyocytes and the underlying mechanisms involved.

**Results:**

The levels of apoptosis-related proteins (PUMA and cleaved caspase-3) were significantly increased in PA + PM-treated H9c2 cells and HFD + PM-treated mouse cardiomyocytes, whereas the antiapoptotic protein Bcl-2 expression was reduced. However, ADSC-CM treatment effectively reduced the PUMA and cleaved caspase-3 expression but increased the Bcl-2 expression. ADSC-CM significantly reduced PA + PM- and HFD + PM-induced cardiomyocyte apoptosis, as detected by the TUNEL assay. RT-qPCR revealed that PA + PM and HFD + PM significantly reduced *miR221/222* levels, whereas ADSC-CM treatment increased *miR221/222* levels. Furthermore, knockout (KO) and transgenic (TG) mice were used to demonstrate that *miR221/222* in ADSC-CM ameliorated cardiac apoptosis that was induced by HFD + PM treatment. Furthermore, PA + PM treatment increased the reactive oxygen species (ROS) production, which triggered mitochondrial fission and contributed to apoptosis. However, ADSC-CM effectively reduced ROS levels and regulated mitochondrial fission, alleviating cellular apoptosis.

**Conclusions:**

Our findings demonstrated that ADSC-CM attenuated PA + PM-induced cardiomyocyte apoptosis by modulating *miR221/222* levels and suppressing ROS production.

**Supplementary Information:**

The online version contains supplementary material available at 10.1186/s13287-025-04381-8.

## Background

Cardiovascular disease (CVD) is the primary cause of mortality globally [1, 2]. Lifestyle choices and environmental factors are crucial for the incidence of CVD. Among them, ordinary lifestyle choices, such as a high-fat diet (HFD), are essential for CVD because they can initiate or aggravate pathophysiological processes, leading to dyslipidemia and obesity in the population [3, 4]. Obesity is closely associated with persistent inflammation and oxidative stress, leading to potential health problems [5, 6]. In addition, air pollution is a serious environmental threat to health worldwide [7]. Many epidemiological and clinical reports have shown that short- and long-term exposure to air pollution, especially particulate matter (PM), significantly enhances the risk of CVDs, including cardiac arrhythmia, hypertension, atherosclerosis, ischemia, and heart failure [8–12]. PM can penetrate the alveolar-capillary barrier, enter the blood, damage the heart, and impair the cardiovascular system [13]. It is generally believed that air pollution causes CVD through oxidative stress. Given that obese patients have poorer oxidative defenses, they were particularly vulnerable to the impact effects of air pollution on cardiovascular health. Additionally, recent research suggests that obese individuals breathe more air per day than normal-weight individuals do and may therefore be more susceptible to the effects of air pollutants [14, 15]. The incidence of CVD resulting from long-term exposure to fine-suspended PM2.5 was closely related to an increased body mass index (BMI), according to a prospective cohort study, suggesting that obese people are more susceptible to the effects of PM2.5 [16, 17]. The mechanisms of the damage to cardiomyocytes caused by PM2.5 exposure and a high-fat diet are unclear, and there is no effective treatment.

Stem cells are undifferentiated cells that possess key characteristics such as differentiation and self-renewal [18]. Adipose-derived stem cells (ADSCs), which belong to mesenchymal stem cells, are derived from human adipose tissue and can differentiate into adipogenic, osteogenic, myogenic, and chondrogenic lineages, as shown by an in vitro study [19]. Because adipose tissue is readily available and preparing ADSCs is relatively easy, ADSCs may be a promising source for cell-based therapies [20]. ADSCs can also repair damaged organs and tissues by secreting large amounts of microRNAs, cytokines, and other bioactive substances. ADSC therapy has been proven to be effective and safe in a variety of animal models [21]. In addition, our previous reports revealed that ADSCs could protect the flap and heart and alleviate the damage caused by ischemia/reperfusion injury [22–24]. However, whether ADSCs can improve cardiomyocyte damage induced by an HFD and PM and the related mechanisms remain to be elucidated. MicroRNA (miRNA) is a small noncoding RNA molecule consisting of 19–25 nucleotides, with highly conserved and tissue-specific expression. They affect gene expression by complete or partial complementary binding to the mRNA of the target gene [25, 26]. MiRNAs affect a variety of biological responses, such as cell apoptosis, proliferation, differentiation, embryogenesis, and tumorigenesis [27]. MiRNAs regulate the activities of cardiomyocytes, endothelial cells, fibroblasts, and smooth muscle cells, which are the constituent cells within the cardiovascular system [28]. MiRNAs are crucial in regulating cardiomyocyte growth and contractility, maintaining the heart rhythm, modulating plaque formation, influencing lipid metabolism, and promoting angiogenesis [27, 29, 30]. Previous studies have reported that *miR221/222* are associated with the progression of cardiovascular pathologies, such as inflammation, apoptosis, angiogenesis, hypertrophy, remodeling, and cardiac fibrosis [31, 32]. Bioinformatics analysis revealed that PUMA is a candidate target of *miR221/222*, and a previous study also revealed that PUMA could regulate apoptosis [33, 34]. Our earlier report demonstrated that enrichment of *miR221/222* in ADSC-CM could alleviate I/R injury [23]. However, the significance of *miR221/222* in the heart during exposure to an HFD and PM remains unclear.

Reactive oxygen species (ROS) are generated as cellular metabolic byproducts and highly reactive oxygen-containing molecules. Several studies have demonstrated that ROS-mediated apoptosis is a critical contributor to the development of CVD [35–37]. Research has also indicated that prolonged exposure to elevated ROS levels may trigger cardiomyocyte dysfunction, leading to cardiac remodeling, apoptosis, necrosis, and fibrosis [38]. Mitochondria are essential organelles in eukaryotic cells and are responsible for ATP synthesis. The mitochondrial electron transport chain is the primary source of ROS during normal metabolic processes. However, excessive intracellular ROS levels can lead to mitochondrial oxidative damage and dysfunction, leading to heart failure, diabetes, neurodegenerative diseases, and cancer [39–41]. Mitochondria in living cells are highly plastic and dynamic organelles that are constantly remodeled through fission and fusion, which affects the number, function, distribution location, and morphology of mitochondria in response to stress. Maintaining the balance between mitochondrial fission and fusion is crucial for the integrity and function of the mitochondrial network. A previous study showed that defects in mitochondrial fusion or fission might affect embryonic development in mice and lead to embryonic lethality [42]. Furthermore, mitochondrial fission has been shown to occur during heart failure [43–46]. Therefore, considering the above factors, the study was designed to explore the impact effects of obesity and air pollution on apoptotic events and to evaluate whether *miR221/222* in ADSC-CM are essential for regulating cardiomyocyte apoptosis by targeting PUMA. In this study, in vitro palmitic acid (PA) treatment and in vivo HFD feeding were used to induce obesity, and PM was used as the air pollution model both in vivo and in vitro. The study revealed that ADSC-CM exerted the cardioprotective function on the reduction in PA + PM-induced cardiomyocyte apoptosis through regulating *miR221/222* and inhibiting ROS production. These findings may provide strategies for preventing and treating HFD + PM-caused cardiac damage in clinical practice.

## Materials and methods

### Cell preparation

The H9c2 cell line, which was derived from embryonic rat cardiac myoblasts, was obtained from the American Type Culture Collection (VA, USA). H9c2 cells were incubated in Dulbecco’s modified Eagle’s medium (DMEM; Gibco, NY, USA) supplemented with 10% (v/v) fetal bovine serum (FBS; Biological Industries, CT, USA), 1% (v/v) L-glutamine (Biological Industries), and 1% penicillin–streptomycin-amphotericin (PSA; Biological Industries). The cells were cultured in a humidified incubator at 37°C and 5% CO_2_.

Human adipose-derived stem cells (ADSCs) were bought from Lonza (Basel, Switzerland) and incubated in DMEM supplemented with 20% (v/v) FBS and 1% PSA. All experiments used cells between passages 3 and 8.

### PM preparation

This study used standard reference material 2786 (SRM 2786; < 4 µm) as the PM. SRM2786 was collected in 2005 from Prague, Czech Republic, and purchased from the Nation Institute of Standards and Technology (MD, USA). To prepare the SRM 2786 stock solution, the pellet was suspended in a cell culture medium at 20 mg/mL concentration and then sonicated three times in cold water for 15 min each. The solution was then preserved at − 20 °C. Before the experiment, the PM suspension was vortexed thoroughly for 1 min to ensure uniform dispersion.

### Preparation of ADSC-conditioned medium (ADSC-CM)

ADSCs were seeded in 10 cm culture dishes at 5 × 10^5^ cells per dish. The culture was continued for 24 h, and the medium was collected and stored at − 20 °C for further studies. ADSC-CM was prepared by mixing the collected culture medium with DMEM in equal proportions (50% of the total volume of the culture medium) immediately before use.

### MTT method

Cell viability was detected using an MTT method. H9c2 cells were plated at a 5 × 10^3^ cells/well density in 96-well plates and incubated overnight. They were then treated with PA (0, 50, 100, or 200 µM) or PM (0, 10, 20, or 40 µg/mL) for 24 h. Subsequently, 0.5 mg/mL MTT solution was added to each well and incubated for 4 h. Dissolve the purple formazan crystals in 150 μL DMSO and measure the absorbance at 570 nm by a microplate reader.

### Western blot analysis

The levels of specific proteins expression were investigated by Western blot. Cells were treated with PA (50 µM), PM (20 µg/mL), or ADSC-CM, and radioimmunoprecipitation assay (RIPA) buffer was used to obtain cell lysates. Equal amounts of protein were separated by 10%, 12%, or 15% SDS-PAGE and transferred to polyvinylidene difluoride (PVDF) membranes. These membranes were treated with 5% blocking buffer at room temperature (RT) for 1 h and then treated overnight with primary antibodies including against *β*-actin (1:5000; A5441, Sigma, MO, USA), GAPDH (1:5000; 60004–1-Ig, Proteintech, IL, USA), PUMA (1:1000; ab9643, Abcam, Cambridge, MA, USA), Bcl-2 (1:1000; 3498S, Cell Signaling Technology, MA, USA), cleaved caspase-3 (cCASP3) (1:1000; #9661, Cell Signaling Technology), SOD1 (1:1000; GTX100554, GeneTex, CA, USA), SOD2 (1:1000; GTX630559, GeneTex), SOD3 (1:1000; GTX81678, GeneTex), p-DRP1 (1:1000; 4494S, Cell Signaling Technology), and FIS1 (1:1000; 10956–1-AP, Proteintech) at 4 °C. These PVDF membranes were washed with Tris-buffered saline with Tween 20 for six times and then treated with a secondary antibody (anti-mouse or anti-rabbit) at RT for 1 h. Bands in immunoblots were examined using the Western Lightning™ chemiluminescence reagent and the Biospectrum 600 imaging system (UVP, CA, USA).

### TdT-mediated dUTP nick end-labeling (TUNEL) assay

According to the instructions, the TUNEL method evaluated apoptosis with a kit (Roche, CA, USA). H9c2 cells were seeded on coverslips (1 × 10^5^) and incubated overnight, followed by treatment with PA (50 µM), PM (20 µg/mL), or ADSC-CM for 24 h. These cells were fixed with 4% paraformaldehyde for 30 min at RT and then incubated with 0.1% Triton X-100 for 5 min at RT. Thereafter, they were labeled with the TUNEL reagent at 37 °C for 1 h, and nuclei were counterstained with DAPI (1 µg/mL) solution for 5 min. After a series of washes, the slides were covered with coverslips. The experimental results were observed and photographed by a fluorescence microscope using appropriate filters.

### Annexin V-FITC/propidium iodide (PI) staining

Apoptosis was examined with an annexin V–FITC/PI detection kit (#640914; BioLegend, Amsterdam, The Netherlands) according to the instructions. H9c2 cells were plated at 2 × 10^5^ cells/well in 6-well plates, cultured overnight, and continued to be treated with PA (50 µM), PM (20 µg/mL), or ADSC-CM for 24 h. Next, the cells were trypsinized, centrifuged, and washed with cold PBS. These cells were subsequently treated with 5 μL of annexin V–FITC and 5 μL of PI at RT for 15 min in the dark. After the above reaction, add 400 μL Annexin V Binding Buffer and immediately detect the results by flow cytometry.

### Real-time quantitative PCR

Samples were collected after the defined treatments. Total RNA was extracted with the TRIzol reagent (Thermo, MA, USA), and the cDNA template was obtained with a TaqMan microRNA reverse transcription kit (Invitrogen, CA, USA) following the manufacturer’s protocol. The *miR221/222* expression levels were analyzed by qPCR using TaqMan Universal PCR Master Mix (Applied Biosystems, CA, USA). The TaqMan microRNA assay kits for *miR221* (000524), *miR222* (002276), and *RNU6B* (001973) were purchased from Applied Biosystems. *MiR221/222* were quantified using the 2^−ΔΔCt^ method, and their levels were expressed as relative amounts after normalization to the *RNU6B* housekeeping gene.

### Transient transfection

H9c2 cells were cultured in a 6-well plate at a density of 2 × 10^5^ cells per well and incubated for 24 h. Then, specific *miR221/222* mimics or inhibitors (Dharmacon, CO, USA) were transfected using Lipofectamine 3000 (Invitrogen) under the manufacturer’s instructions. The levels of apoptosis of *miR221/222*-overexpressing or *miR221/222*-knockdown cells were evaluated.

#### Animal experiments

Male C57BL/6 (B6) mice, miR221/222 knockout (KO) mice, and miR221/222 transgenic (TG) mice (8–12 weeks old, weighing 20 g) were used. B6 mice were purchased from the Laboratory Animal Center of National Taiwan University. This mouse strain was provided by the Jackson Laboratory (Stock No. 000664, Bar Harbor, Maine, USA). MiR-221/222 KO mice were generated by deleting the X-linked miR-221/222 gene and backcrossed on a B6 background for 10 generations. All experimental procedures were conducted under the guidelines of the Institutional Animal Care and Use Committee (IACUC; No. 20210334), National Taiwan University College of Medicine and College of Laboratory Animal Center. All animals were maintained under specific pathogen-free (SPF) conditions with individually ventilated cages, a 12-h light/dark cycle, and free access to food and water. B6 and KO mice were randomly divided into four groups (6–10 in each group): (1) the control group (Con); (2) the high-fat diet + particulate matter (HFD + PM) group; (3) the high-fat diet + particulate matter + ADSC-CM (HFD + PM + CM) group; and (4) the ADSC-CM (CM) group. TG mice were randomly divided into two groups (6 in each group): (1) the Con group and (2) the HFD + PM group. A total of 76 mice were used in this study. The number of mice in each group was greater than or equal to 6, allowing for biostatistics. A single mouse was considered the experimental unit for this study. The mice were fed a regular diet or an HFD (DIO rodent purified diet containing 60% energy from fat-blue; 58Y1) for 4 weeks. In the HFD + PM and HFD + PM + CM groups, intratracheal injections of PM (10 mg/kg) were administered at the 3rd and 4th weeks. Briefly, mouse was anesthetized using 2% isoflurane, and the trachea was carefully exposed. The PM was applied to the anterior wall using an insulin syringe held at a 45° angle to minimize posterior wall damage. After the injection, the trachea and wound were sutured. The mouse was returned to cages after regaining consciousness. The mice in the HFD + PM + CM and CM groups were injected with CM through the tail vein at the 3rd and 4th weeks. After 4 weeks, mice were anesthetized with 2% isoflurane until they were utterly unconscious and then subjected to cervical dislocation to ensure that euthanasia was performed humanely and quickly. Randomising the order of examination reduced bias. Moreover, objective measures were employed, including qPCR for miRNA levels, Western blot for protein expression, and TUNEL-positive cell counting for apoptosis evaluation, to minimize potential bias. The work is reported in line with the ARRIVE guidelines 2.0.

#### Cardiac function assessment

Cardiac functional and structural examinations were conducted using a high-resolution small animal ultrasound system (Prospect; S-Sharp, Taiwan) with a 40-MHz single-element sensor. M-mode imaging of the left ventricular short-axis view and mitral valve Doppler echocardiography were performed; three consecutive cycles and the average of three measurements were averaged. The fractional shortening (FS), ejection fraction (EF), left ventricular posterior wall thickness in diastole (LVPWd), left ventricular posterior wall thickness in systole (LVPWs), left ventricular isovolumetric contraction time (IVCT), left ventricular isovolumetric relaxation time (IVRT), mitral E/A ratio, and stroke volume (SV) were calculated using standard formulas.

#### Histopathological staining

Mouse hearts were collected and fixed with 4% paraformaldehyde. Cardiac tissues were embedded in paraffin and subsequently cut at 5 μm thickness. The sections were conducted to hematoxylin and eosin staining, TUNEL staining, or PUMA immunohistochemical staining.

#### MitoSOX Red staining

MitoSOX Red (Invitrogen) was used to detect ROS in mitochondria. In this experiment, H9c2 cells were plated in 4-well chamber slides (5 × 10^4^ cells/well), cultured overnight, and then incubated with PA (50 µM), PM (20 µg/mL), or ADSC-CM. After 24 h, the media was removed, and these cells were incubated with a 5 µM MitoSOX reagent at 37 °C for 10 min in the dark. Nuclei were stained with Hoechst. After washing the samples with PBS, fluorescence detection was performed using a fluorescence microscope (Leica, Germany) with an excitation wavelength (510 nm) and an emission wavelength (580 nm). For flow cytometry experiments, H9c2 cells were incubated overnight at 2 × 10^5^ cells/well in 6-well plates following the same protocol. They were trypsinized and examined on an LSRFortessa flow cytometer (BD Biosciences, CA, USA).

#### JC-1 staining

The fluorescent probe 5,5′,6,6′-tetrachloro-1,1′,3,3′-tetraethylbenzimidazolocarbocyanine iodide (JC-1; BD Biosciences) was used to examine the mitochondrial membrane potential (Δψm). H9c2 cells were plated overnight in 4-well chamber slides (5 × 10^4^ cells/well). Cells were incubated with PA, PM, or ADSC-CM for 24 h, the medium was removed, and then the cells were treated with 1 μg/mL JC-1 solution for 30 min at 37 °C in the dark. The images were photographed by fluorescent microscopy. The monomeric form of JC-1 emits green, whereas the aggregated form becomes red. For flow cytometry experiments, H9c2 cells were incubated overnight at a density of 2 × 10^5^ cells per well in 6-well plates following the same protocol. The cells were trypsinized and examined by an LSRFortessa flow cytometer.

#### ATP measurement

According to the guidelines, the ATP levels were analyzed using an ATP assay kit (Thermo). After the indicated treatments, H9c2 cells were trypsinized and centrifuged at 900 × g for 10 min. The pellets were then suspended in RIPA buffer and incubated for 20 min at 4 °C. The solution was subsequently centrifuged at 17,000 × g for 20 min at 4 °C. The supernatants were collected. Finally, 10 µL of the supernatant was mixed with 90 µL of ATP assay buffer to determine ATP levels, and luminescence was examined by a microplate reader. The ATP concentrations were normalized and assessed via a standard curve, and changes were compared with those in the control group.

#### Assessment of mitochondrial length

Fluorescence staining for mitochondrial identification was performed by using MitoTracker Red (Thermo). After H9c2 cells were subjected to specific treatments, they were incubated with a 400 nM MitoTracker Red solution for 45 min at 37 °C in the dark. Subsequently, images were obtained by total internal reflection fluorescence microscopy (TIRFM; Zeiss) combined with DIC optics. The mitochondrial length was quantified through the ImageJ software.

#### Statistical analysis

The data are expressed as the means ± SEM and were examined using a one-way analysis of variance (ANOVA) with Fisher’s post hoc test. *P* values < 0.05 indicated significant differences for all analyses.

## Results

### Combined treatment with PA and PM significantly increases cell apoptosis, whereas ADSC-CM reduces this effect

**Fig. 1:**
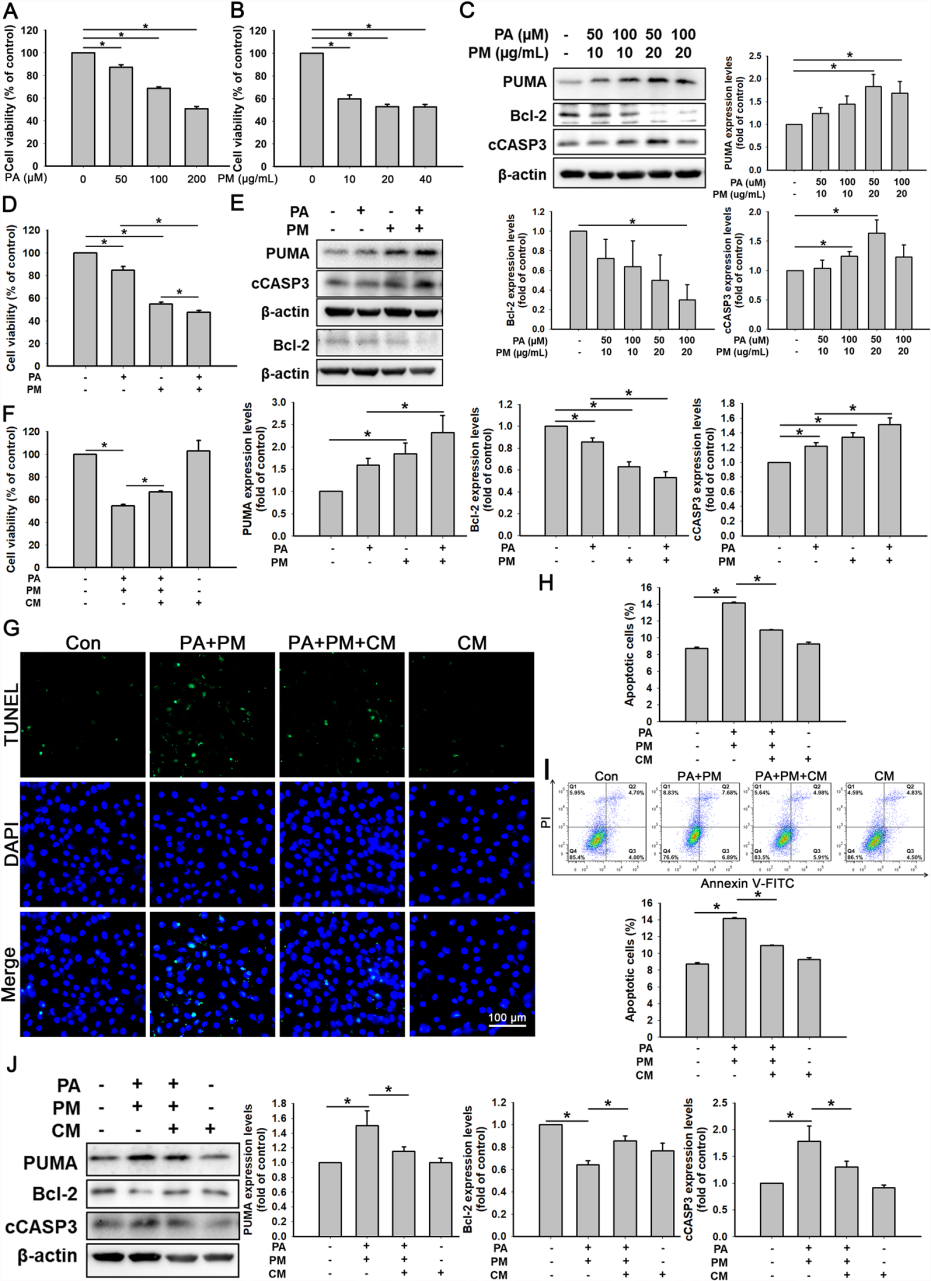
Effects of ADSC-CM on PA + PM-induced H9c2 cell apoptosis. **A** H9c2 cells were treated with PA at various concentration (0, 50, 100, and 200 µM) for 24 h. An MTT method measured cell viability. **B** H9c2 cells were incubated with various doses (0, 10, 20, and 40 µg/mL) of PM for 24 h. An MTT method measured cell viability. **C** H9c2 cells were co-treated with the defined concentratin of PA and PM for 24 h. The expression levels of PUMA, Bcl-2, and cCASP3 were checked by Western blot. **D** H9c2 cells were incubated with PA (50 µM), PM (20 µg/mL), or PA (50 µM) + PM (20 µg/mL) for 24 h. An MTT method measured cell viability. **E** H9c2 cells were treated with PA (50 µM), PM (20 µg/mL), or PA (50 µM) + PM (20 µg/mL) for 24 h. **F** H9c2 cells were treated with 50 µM PA, 20 µg/mL PM, and ADSC-CM for 24 h. An MTT method measured cell viability. **G** H9c2 cells were treated with 50 µM PA, 20 µg/mL PM, and ADSC-CM for 24 h and then examined for apoptosis via a TUNEL method (TUNEL-positive cells: green; nuclei: blue). Scale bar = 100 μm. **H** The bar graph depicts the quantitative analysis results of counting the number of TUNEL-positive cells. **I** H9c2 cells were incubated with 50 µM PA, 20 µg/mL PM, and ADSC-CM for 24 h. Cell death was investigated by detecting annexin V-FITC and propidium iodide (PI) staining and analyzing by flow cytometry. **J** H9c2 cells were incubated with 50 µM PA, 20 µg/mL PM, and ADSC-CM for 24 h. Western blot examined the PUMA, Bcl-2, and cCASP3 expression levels. Experiments were conducted at least five times, and the quantitative data were presented as mean ± SEM. *P < 0.05 is defined as statistically significant. PA: palmitic acid; PM: particulate matter; CM: ADSC-CM; cCASP3: cleaved caspase-3

PA remarkably decreased cell viability in a concentration-dependent manner (50, 100, and 200 μM) by the MTT assay (Fig. 1A). PM also significantly reduced cell viability at different doses (10, 20, and 40 μg/mL; Fig. 1B). Furthermore, the combined treatment with 50 µM PA and 20 µg/mL PM significantly increased the expression levels of the apoptosis-related proteins PUMA and cCASP3, while the antiapoptotic protein Bcl-2 expression was decreased (Fig. 1C; Figure S1 for the full-length blot). To further determine whether combined stimulation with 50 µM PA and 20 µg/mL PM has an additive effect on H9c2 cell apoptosis, we measured cell viability and protein levels of PUMA, Bcl-2, and cCASP3. As shown in Fig. 1D, compared with PA or PM alone, H9c2 cells co-treated with PA and PM for 24 h considerably reduced the cell viability. Furthermore, PA + PM increased the levels of PUMA and cCASP3 and reduced the expression of Bcl-2 (Fig. 1E; full-length blots are provided in Figure S2).

To detect the impact effect of ADSC-CM on PA + PM-treated H9c2 cells, these cells were further treated with ADSC-CM under the cotreatment of PA and PM for 24 h. As shown in Fig. 1F, ADSC-CM significantly restored cell viability. The TUNEL assay indicated that PA + PM resulted in a remarkable increase in apoptotic cells; however, ADSC-CM reduced PA + PM-induced apoptosis (Fig. 1G; quantitative analysis of TUNEL-positive cells is shown in Fig. 1H). As shown in Fig. 1I, increases in early (annexin V^+^ and PI^−^) and late (annexin V^+^and PI^−^) cell apoptosis were observed in PA + PM-treated H9c2 cells. In contrast, ADSC-CM reduced both early and late apoptosis. In Fig. 1J (full-length blots in Fig. S3), PA + PM increased the expression levels of PUMA and cCASP3 and reduced the Bcl-2 expression. In contrast, ADSC-CM decreased the PUMA and cCASP3 levels and increased the Bcl-2 expression levels. These above results showed that PA and PM induced H9c2 cell apoptosis, whereas ADSC-CM protected H9c2 cells from PA- and PM-induced apoptosis.

### ADSC-CM protects cardiomyocytes from PA + PM-induced apoptosis via *miR221/222*

**Fig. 2:**
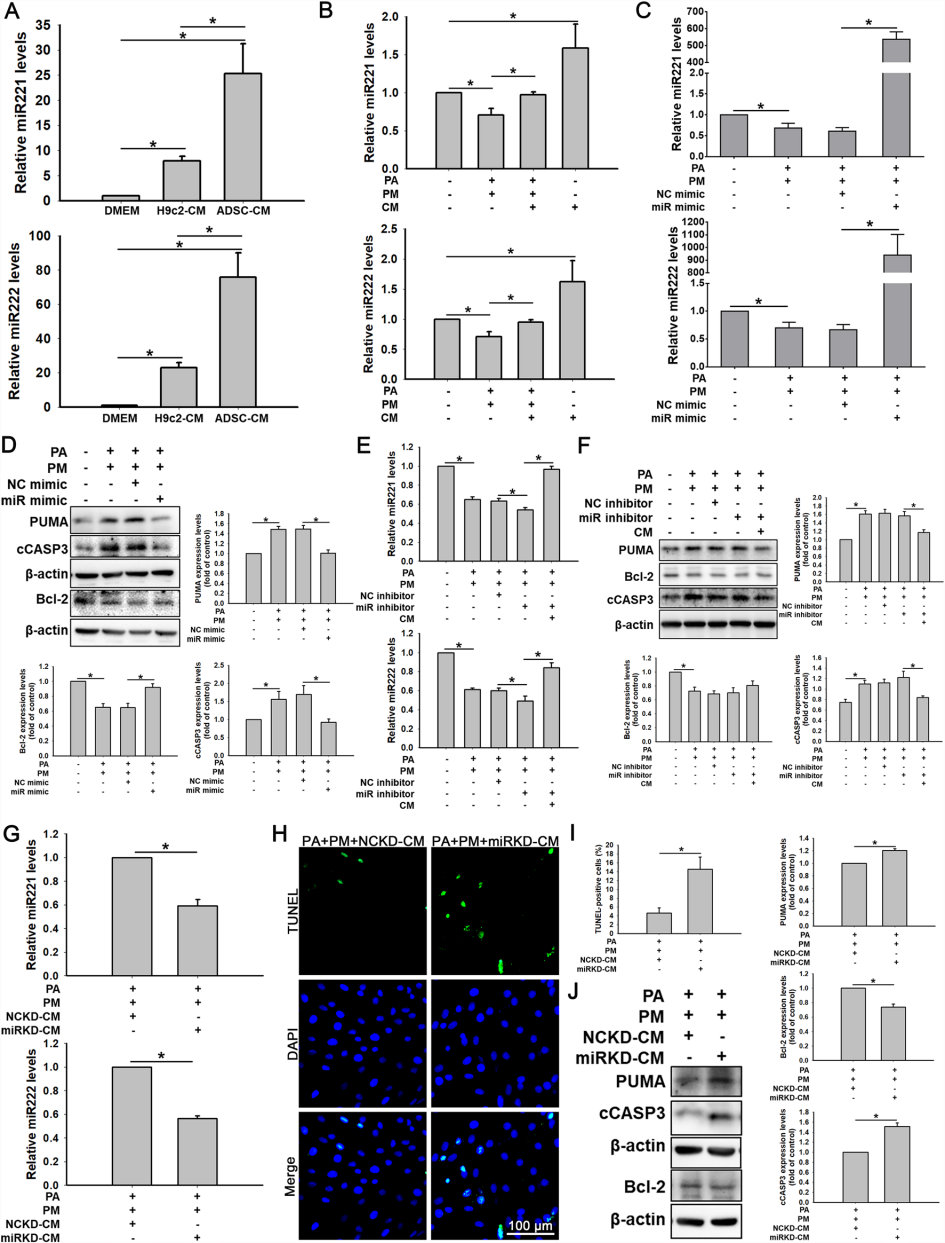
Effects of ADSC-CM on PA + PM-induced H9c2 cell apoptosis through *miR221/222*. **A** *MiR221/222* levels were determined in DMEM, H9c2-CM, and ADSC-CM by qPCR. **B** H9c2 cells were incubated with 50 µM PA, 20 µg/mL PM, and ADSC-CM for 24 h. The expression levels of *miR221/222* were evaluated by qPCR. **C**, **D** H9c2 cells were transfected with the corresponding negative control (NC) or *miR221/222* mimics for 24 h and then incubated with 50 µM PA and 20 µg/mL PM for 24 h. *MiR221/222* expression levels were examined by qPCR (**C**), and PUMA, Bcl-2, and cCASP3 protein expression levels were investigated by Western blot (**D**). **E**, **F** H9c2 cells were transfected with a scramble sequence or *miR221/222* inhibitors for 24 h and then treated with 50 µM PA, 20 µg/mL PM, and ADSC-CM for 24 h. The *miR221/222* expression levels were assessed by qPCR (**E**). PUMA, Bcl-2, and cCASP3 expression levels were investigated by Western blot (**F**). **G** ADSCs were transfected with the corresponding negative control (NC) or *miR221/222* inhibitors for 24 h. These cells were then incubated in a standard culture medium for another 24 h, and NCKD-CM and miRKD-CM were collected, respectively. The levels of *miR221/222* were measured by qPCR. **H** Apoptosis was assessed via a TUNEL method (TUNEL-positive cells: green; nuclei: blue). Scale bar = 100 μm. **I** Quantification data of TUNEL-positive cells is shown in the bar graphs. **J** Western blot measures the PUMA, Bcl-2, and cCASP3 expression levels. Experiments were conducted at least five times, and the quantitative data were presented as mean ± SEM. *P < 0.05 is defined as statistically significant. NC mimic: negative control mimic; miR mimic: *miR221/222* mimic; NC inhibitor: negative control inhibitor; miR inhibitor: *miR221/222* inhibitor; NCKD-CM: CM obtained after the knockdown of the negative control in ADSCs; miRKD-CM: CM obtained after the knockdown of *miR221/222* in ADSCs

The above results confirmed that ADSC-CM could reduce PA- and PM-induced cardiomyocyte apoptosis, but the specific mechanism remains unclear. An earlier study revealed that ADSC-CM is enriched in *miR221/222*, potentially protecting cardiomyocytes from hypoxia/reperfusion-induced injury [23]. To confirm that ADSC-CM contained high amounts of *miR221/222*, CM was collected from H9c2 cells or ADSCs, and DMEM was used as a control. Compared with DMEM and H9c2-CM, ADSC-CM contained more *miR221/222*, as shown by the qPCR assay (Fig. 2A). PA + PM significantly reduced *miR221/222* levels, whereas ADSC-CM reduced this effect (Fig. 2B). Bioinformatics analysis of *miR221/222* was performed to predict their gene targets, particularly PUMA as a potential target, and the study aimed to understand the roles of *miR221/222* in PA + PM-induced apoptosis. In Fig. 2C, the amounts of *miR221/222* in H9c2 cells transfected with *miR221/222* mimics were remarkably higher than those in the negative control (NC) miRNA group under both PA and PM conditions. Under combined treatment with PA and PM, cells transfected with the *miR221/222* mimics presented significantly decreased PUMA and cCASP3 levels and increased Bcl-2 expression (Fig. 2D; full-length blots are shown in Fig. S4). To further elucidate the roles of *miR221/222* in PA + PM-induced apoptosis, H9c2 cells were transfected with the *miR221/222* inhibitor or negative control inhibitor. To effectively inhibit *miR221/222* expression, we used PA + PM stimulation to reduce *miR221/222* expression and inhibited endogenous *miR221/222* expression by transfection, as shown in Fig. 2E. PA + PM considerably decreased the levels of *miR221/222* expression compared with control treatment. The combination of the *miR221/222* inhibitor and PA + PM treatment (PA + PM + miR221/222 inhibitor) significantly decreased the *miR221/222* levels; however, ADSC-CM treatment increased the *miR221/222* levels. Subsequently, this study used Western blot to analyze the changes in the levels of apoptosis-related protein expression under the same conditions. As shown in Fig. 2F (full-length blots in Fig. S5), the PA + PM or PA + PM + *miR221/222* inhibitor considerably increased the PUMA and cCASP3 expression levels and reduced the Bcl-2 expression. In contrast, ADSC-CM decreased the PUMA and cCASP3 expression levels and increased the Bcl2 expression. Herein, we demonstrated that *miR221/222* in CM effectively increased *miR221/222* levels, whereas PA + PM treatment and the transfection with the *miR221/222* inhibitors decreased *miR221/222* levels. These results showed that PUMA was negatively regulated by *miR221/222* in PA + PM-treated H9c2 cells. *MiR221/222* inhibits apoptosis by targeting PUMA to further regulate downstream apoptotic pathways.

Since ADSC-CM contains large amounts of secreted growth factors, cytokines, and miRNAs, to determine whether *miR221/222* or other factors in ADSC-CM affect apoptosis, we treated ADSCs with *miR221/222* inhibitors or negative control miRNA inhibitors and collected miRKD-CM and NCKD-CM, respectively. In combination with PA and PM treatment, the levels of *miR221/222* in H9c2 cells incubated with miRKD-CM were considerably lower than those in cells treated with NCKD-CM, as provided in Fig. 2G. The TUNEL method indicated that apoptotic cells increased markedly after miRKD-CM treatment (Fig. 2H and I). Compared with NCKD-CM treatment, miRKD-CM treatment also considerably increased PUMA and cCASP3 levels and decreased Bcl-2 expression levels (Fig. 2J; full-length blots are presented in Fig. S6). These data suggest that ADSC-CM administration reduces PA + PM-induced apoptosis, mainly because ADSC-CM contains large amounts of *miR221/222*. These results also revealed that *miR221/222* were potent regulators of cardiomyocyte apoptosis.

### ADSC-CM protects B6 and KO mice from HFD + PM-induced apoptosis via *miR221/222*

**Fig. 3:**
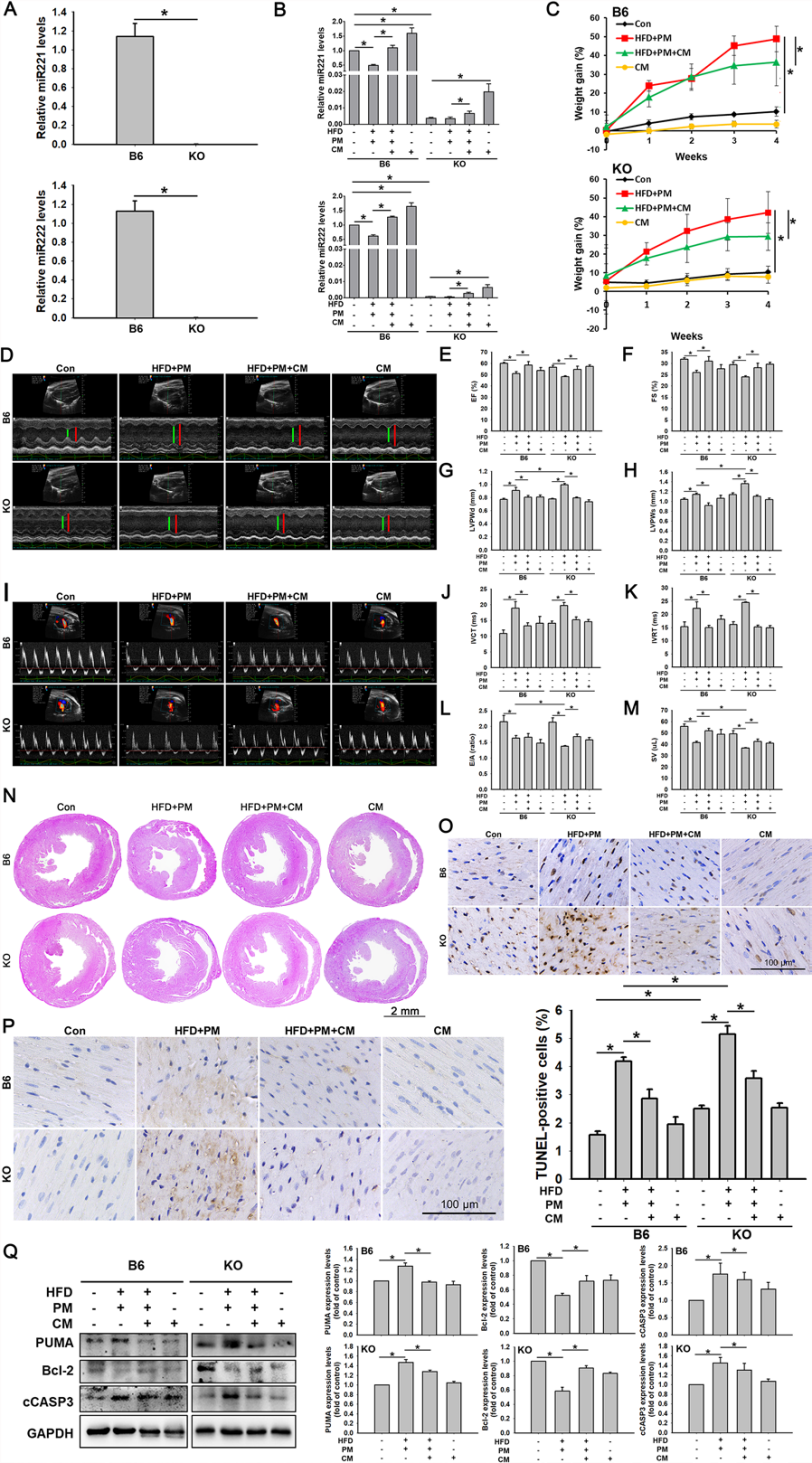
Effects of ADSC-CM on high-fat diet (HFD) + PM-induced apoptosis in B6 and KO mice via *miR221/222*. **A** Determination of *miR221/222* levels in heart tissues from B6 and KO mice by qPCR. **B** Mice were fed a HFD for 4 weeks and were injected with or without PM and CM at the third and fourth weeks. The levels of *miR221/222* in heart tissues were determined by qPCR. **C** Mice were weighed before the start of the experiment and weekly thereafter. **D** Representative photographs of transthoracic echocardiographic morphology in M mode. **E**–**H** Measurements of the ejection fraction (EF), fractional shortening (FS), diastolic left ventricular posterior wall thickness (LVPWd), and systolic left ventricular posterior wall thickness (LVPWs). **I** Representative images of mitral valve waveforms from transthoracic echocardiography. **J**–**M** Measurements of the left ventricular isovolumetric contraction time (IVCT), left ventricular isovolumetric relaxation time (IVRT), mitral valve E/A ratio, and stroke volume (SV). **N** Representative images of HE-stained cardiac tissue (scale bar = 2 mm). **O** Apoptosis was assessed in cardiac tissue using a TUNEL assay. The positive reaction product is brown. The nuclei were counterstained with hematoxylin solution. **P** The location of PUMA expression in cardiac tissue was investigated by immunohistochemical staining. The positive reaction product is brown. The nuclei were counterstained with hematoxylin solution. **Q** PUMA, Bcl-2, and cCASP3 expression levels were examined by Western blot. Experiments were conducted at least six times, and the quantitative data were presented as mean ± SEM. *P < 0.05 is defined as statistically significant. B6: C57B/L6 mice; KO: *miR221/222* knockout mice; HFD: high-fat diet

The study used C57BL/6 (B6) and *miR221/222* knockout (KO) mouse strains to investigate the effects of *miR221/222* on HFD- and PM-induced cardiomyocyte apoptosis in vivo. Initially, the absence of *miR221/222* expression in KO mice was demonstrated using qPCR (Fig. 3A). B6 and KO mice were divided into control, HFD + PM, HFD + PM + ADSC-CM, and ADSC-CM groups. As shown in Fig. 3B, in B6 mice, the levels of *miR221/222* were considerably lower in the HFD + PM group than those in the control group. Compared with the HFD + PM group, the expression levels of *miR221/222* in the HFD + PM + ADSC-CM group were remarkably increased. In KO mice, we also found that the levels of *miR221/222* increased under ADSC-CM treatment in both the HFD + PM + ADSC-CM and ADSC-CM groups. These findings confirmed that ADSC-CM contained *miR221/222*, which could effectively persist in KO mice. We also studied the impact of the HFD on the body weight of mice. Figure 3C shows that after 4 weeks of feeding, the body weights of the animals in the HFD + PM group were significantly greater than those in the control group, whereas those in the ADSC-CM group were lower. Additionally, we used echocardiographic analysis to evaluate cardiac function (Fig. 3D). An M-mode ultrasound cardiac examination revealed that in the HFD + PM group, the EF and FS were lower and the LVPWd and LVPWs were greater than those in the Con group. In contrast, the EF and FS were greater in the HFD + PM + ADSC-CM group than those in the HFD + PM group. The LVPWd and LVPWs were lower in the HFD + PM + ADSC-CM group than those in the HFD + PM group (Fig. 3E–H). Doppler echocardiography was used to examine diastolic function (Fig. 3I). Compared with those in the Con group, the IVCT and IVRT increased and the mitral valve E/A ratio and SV decreased in the HFD + PM group. In contrast, IVCT and IVRT were decreased, and mitral E/A ratio and SV were increased in the HFD + PM + ADSC-CM group compared to those in the HFD + PM group (Fig. 3J–M). The experimental results suggest that combined HFD and PM treatment impairs cardiac function in mice; however, ADSC-CM treatment improves this function. Next, hematoxylin and eosin staining examined the morphology of the cardiac tissues histologically (Fig. 3N). We observed no differences among the Con, HFD + PM, HFD + PM + CM, and CM groups of B6 or KO mice. We used TUNEL analysis to determine whether ADSC-CM affects HFD + PM-induced cell apoptosis. In the HFD + PM group, more apoptotic cells were observed; however, ADSC-CM significantly reduced the number of HFD + PM-induced apoptotic cells in the B6 and KO mice (Fig. 3O). In addition, a comparison between the B6 and KO mice revealed higher numbers of TUNEL-positive cells in the Con and HFD + PM groups of the KO mice than in those of the B6 mice. To confirm the relationship between *miR221/222* and PUMA expression, we used immunohistochemistry. As shown in Fig. 3P, in the B6 and KO mice, PUMA expression was stronger in the HFD + PM groups than in the HFD + PM + ADSC-CM groups. Comparison between the respective HFD + PM groups showed that the PUMA expression in the KO mice was greater than that in the B6 mice. PUMA and cCASP3 expression levels were increased, and the Bcl-2 expression levels were reduced in the HFD + PM groups. In contrast, ADSC-CM reduced these effects (Fig. 3Q; full-length blots are presented in Figure S7). These results suggest that HFD- and PM-induced myocardial injury may be more severe in the absence of *miR221/222*. HFD + PM can cause cardiomyocyte apoptosis by downregulating *miR221/222*.

### *MiR221/222* TG mice presented fewer apoptotic cells under HFD and PM conditions

**Fig. 4:**
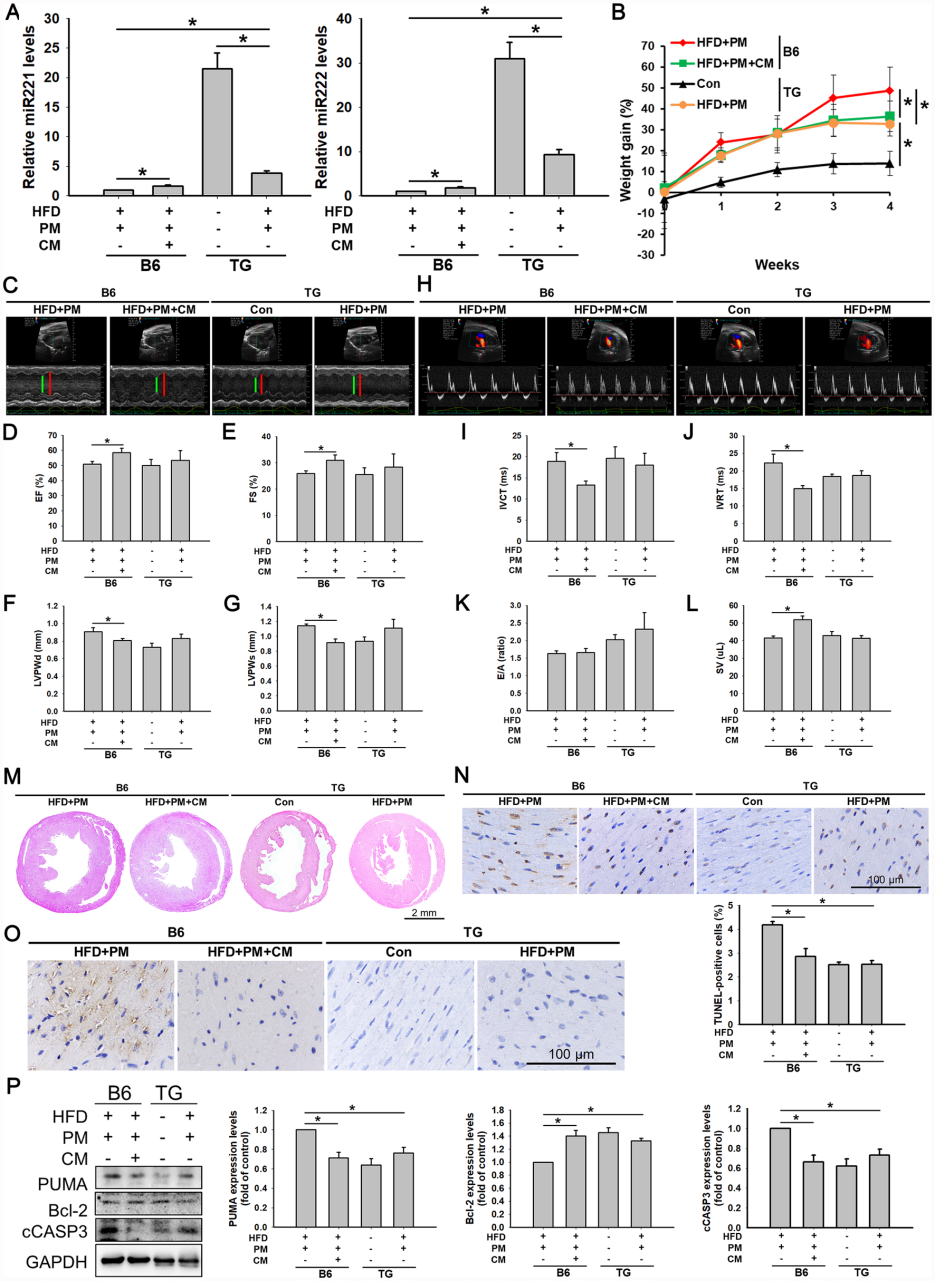
Effects of *miR221/222* on apoptosis of high-fat diet (HFD) + PM-treated TG mice. **A** Mice were fed an HFD for 4 weeks and injected with or without PM and CM at the third and fourth weeks. The levels of *miR221/222* were determined by qPCR. **B** Mice were weighed before the start of the experiment and weekly thereafter. **C** Representative M-mode images of transthoracic echocardiography. **D**–**G** Measurements of the EF, FS, LVPWd, and LVPWs. **H** Representative images of mitral valve waveforms from transthoracic echocardiography. **I**–**L** Measurements of the IVCT, IVRT, mitral E/A ratio, and SV. **M** Representative images of HE-stained cardiac tissues (scale bar = 2 mm). **N** Apoptosis was examined in cardiac tissues using a TUNEL assay. The positive reaction product is brown. The nucleus was counterstained with hematoxylin solution. Scale bar = 100 µm. **O** Immunohistochemistry was used to detect the location of PUMA expression in cardiac tissue. The positive reaction product is brown. The nuclei were counterstained with hematoxylin solution. **P** Western blot examined the PUMA, Bcl-2, and cCASP3 expression levels. These experiments were conducted at least six times, and the quantitative data were presented as mean ± SEM. *P < 0.05 is defined as statistically significant. TG: *miR221/222* transgenic mice

The functional involvement of *miR221/222* expression in the effects of ADSC-CM on HFD- and PM-induced cardiomyocyte apoptosis was further evaluated in the *miR221/222* TG mice. The TG mice were divided into two experimental groups, receiving or not receiving combined HFD and PM treatment. B6 mice served as the control group and were divided into the HFD + PM and HFD + PM + ADSC-CM groups. The qPCR results revealed that in the TG mice, the levels of *miR221/222* were greater without HFD and PM treatment, whereas HFD and PM treatment reduced their expression. Importantly, *miR221/222* levels in TG mice were remarkably higher than those in B6 mice that received the same HFD and PM treatment (Fig. 4A). We then monitored changes in the body weight, as shown in Fig. 4B. After 4 weeks of experimental feeding, the body weights of the B6 and TG mice treated with the HFD and PM increased significantly. Subsequently, the ventricular function was assessed with echocardiography (Fig. 4C). By comparing the differences between the HFD + PM groups of the B6 and TG mice in M mode, we found that the EF and FS values of the TG mice tended to increase compared with those of the B6 mice. However, the LVPWd and LVPWs tended to decrease (Fig. 4D–G). Mitral valve Doppler ultrasound cardiac examination revealed that compared with those of the B6 mice, the IVCT and IVRT values of the TG mice were lower, whereas the E/A value was greater. There was no apparent difference in the SV indices between the HFD + PM groups of the B6 and TG mice (Fig. 4H–L). These results demonstrated that elevated levels of *miR221/222* mitigated the deleterious effects of HFD and PM on cardiac diastolic and systolic function in mice.

Heart sections were subsequently histologically examined using hematoxylin and eosin staining. As shown in Fig. 4M, no apparent differences were observed between the B6 and TG mouse groups. However, evaluation of apoptosis in cardiac tissue using a TUNEL staining assay revealed that in the respective HFD + PM groups, the percentage of apoptotic cells in the TG mice was markedly lower than that in the B6 mice (Fig. 4N). Furthermore, we evaluated the expression of PUMA using immunohistochemistry to determine its relationship with *miR221/222* levels. Upon HFD and PM treatment, PUMA expression in the TG mice was remarkably lower than that in the B6 mice, as shown in Fig. 4O. The levels of PUMA and cCASP3 were consistently reduced. In contrast, the expression level of Bcl-2 was more significant in the HFD + PM group of the TG mice than in that of the B6 mice (Fig. 4P; full-length blots are presented in Figure S8). These results show that *miR221/222* can inhibit PUMA expression and alleviate the harmful impact of HFD and PM on cardiomyocyte apoptosis. Furthermore, *miR221/222* in ADSC-CM are intrinsically involved in reducing HFD- and PM-induced cardiomyopathy.

### ADSC-CM attenuated PA- and PM-induced mitochondrial ROS (mROS) production in H9c2 cells

**Fig. 5:**
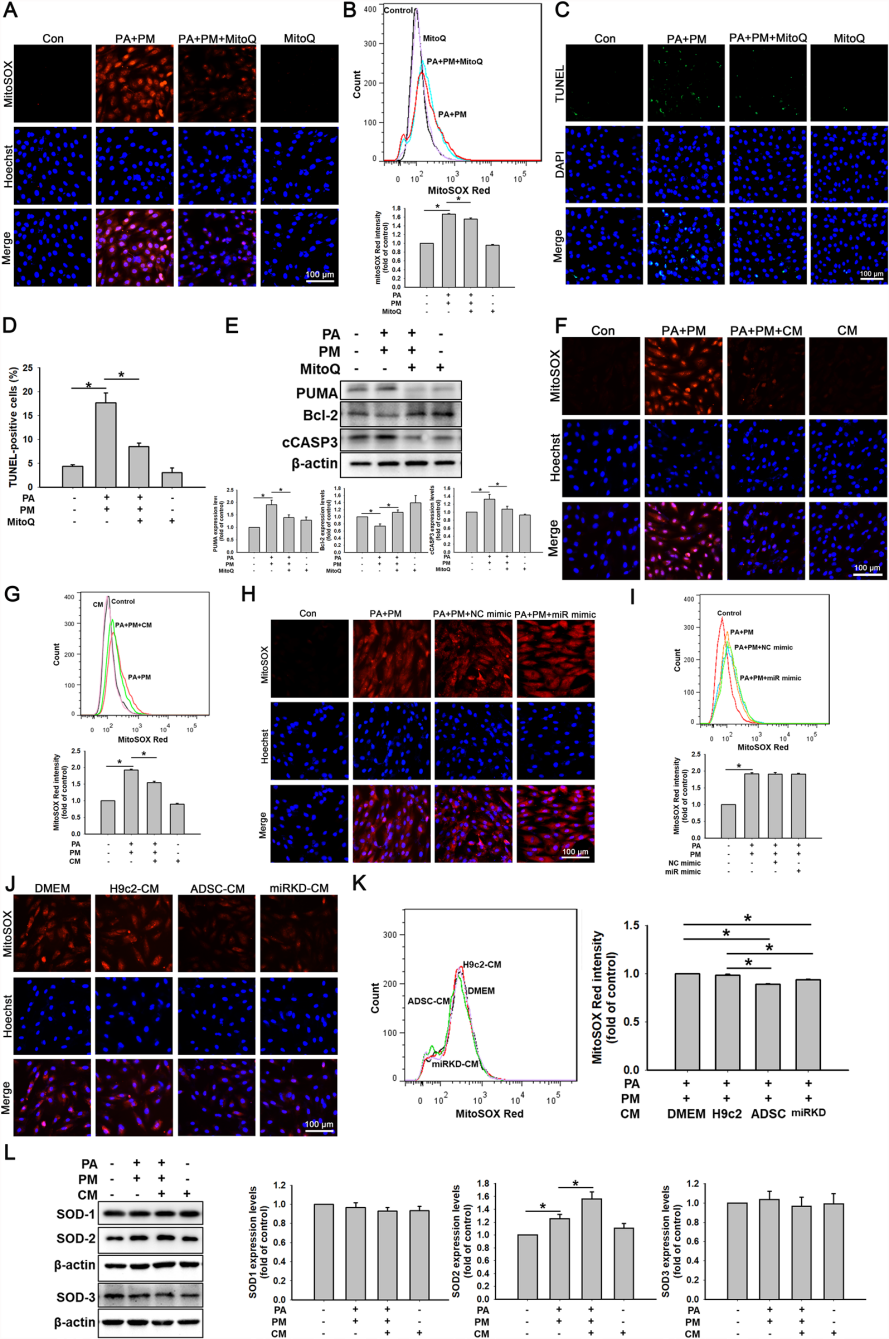
Effects of ADSC-CM on ROS production in PA- and PM-treated H9c2 cells. **A**–**E** H9c2 cells were incubated with 50 µM PA, 20 µg/mL PM, and 100 nM MitoQ for 24 h. MitoSOX Red staining measures mitochondrial ROS levels using fluorescent microscopy (**A**) and flow cytometry (**B**). Apoptosis was detected using a TUNEL method (TUNEL-positive cells: green; nuclei: blue) (**C**). The statistical graph depicts the quantification of TUNEL-positive cell count (**D**). The PUMA, Bcl-2, and cCASP3 expression levels were investigated by Western blot (**E**). **F**, **G** H9c2 cells were treated with 50 µM PA, 20 µg/mL PM, and CM for 24 h. They were then treated with MitoSOX Red and detected by fluorescent microscopy (**F**) and flow cytometry (**G**). **H**, **I** H9c2 cells were incubated with the corresponding negative control (NC) or *miR221/222* mimics for 24 h and then incubated with 50 µM PA and 20 µg/mL PM for 24 h. They were treated with MitoSOX Red and checked via fluorescence microscopy (**H**) or flow cytometry (**I**). **J**, **K** ADSCs were transfected with *miR221/222* inhibitors and then miRKD-CMs were collected. H9c2 cells were co-treated with 50 µM PA, 20 µg/mL PM, and DMEM or CM from H9c2, ADSCs, and miRKD (H9c2-CM, ADSC-CM, and miRKD-CM) for 24 h. The cells were then treated with MitoSOX Red and detected by fluorescent microscopy (**J**) and flow cytometry (**K**). **L** The SOD1, SOD2, and SOD3 expression levels were analyzed using Western Blot. The experiments were conducted at least five times, and the quantitative data were presented as mean ± SEM. *P < 0.05 is defined as statistically significant. Scale bar = 100 µm

Overproduction of ROS and excessive oxidative stress are closely related to the pathogenesis of CVD. The previous study showed that concomitant HFD intake and PM2.5 exposure exacerbated cardiac fibrosis through ROS production in C57BL6/J mice [47]. In this study, MitoSOX Red staining revealed that PA + PM increased the mROS production, as shown through fluorescent microscopy and flow cytometry (Fig. 5A and B). Mitoquinone (MitoQ), a mitochondrion-targeted antioxidant, reduced PA- and PM-induced ROS production in H9c2 cells. A TUNEL method was conducted to determine whether lowering ROS production could ameliorate PA- and PM-induced H9c2 cell death. In the PA and PM combined treatment group, MitoQ significantly reduced H9c2 cell apoptosis (Fig. 5C). The bar graph in Fig. 5D shows the results of the quantitative analysis of the number of TUNEL-positive cells. MitoQ treatment also decreased the PUMA and cCASP3 levels and increased the Bcl-2 expression levels (Fig. 5E; full-length blots are presented in Figure S9). These results indicate that a reduction in ROS production can reduce PA- and PM-induced cell death.

Compared with that in the PA + PM treatment group, ROS production was decreased by ADSC-CM treatment (Fig. 5F and G). ADSC-CM reduced PA- and PM-induced apoptosis by inhibiting ROS production. These results confirmed that ADSC-CM contained high levels of *miR221/222*, which can hinder PA- and PM-induced cardiomyocyte apoptosis. To gain further insights into the relationship between *miR221/222* and ROS levels in cardiomyocyte apoptosis, cells were transfected with *miR221/222* mimics to determine whether they inhibit PA + PM-induced ROS production. As shown in Fig. 5H and I, the overexpression of *miR221/222* did not inhibit PA- and PM-induced ROS production. Conditioned media from cultures of H9c2 cells did not reduce PA and PM-induced mROS production in H9c2 cells. In contrast, ADSC-CM or miRKD-CM treatment reduced it (Fig. 5J and K). The findings indicate that the reduction of PA + PM-induced ROS production by ADSC-CM may be independent of *miR221/222*. On the other hand, PA + PM may promote cardiomyocyte apoptosis through two pathways, namely, by increasing ROS production and reducing *miR221/222* expression. Therefore, we believe that ADSC-CM increases the expression level of *miR221/222* and inhibits the production of ROS, thereby decreasing PA + PM-induced cardiomyocyte apoptosis. Furthermore, intracellular ROS levels are regulated by the balance between ROS production and the levels of antioxidant enzymes, such as SOD1, SOD2, and SOD3 expression. Therefore, we also studied SOD1, SOD2, and SOD3 expression levels in PA + PM-treated H9c2 cells. Figure 5L (full-length blots are shown in Figure S10) shows that the level of the antioxidant enzyme SOD2 was increased in H9c2 cells treated with PA + PM. Furthermore, ADSC-CM treatment significantly increased the expression of SOD2 under PA + PM conditions. In summary, ADSC-CM can reduce ROS production by increasing the expression of the mitochondrial enzyme SOD2, further reducing PA + PM-induced cell death.

### ADSC-CM reduced PA- and PM-induced mitochondrial fission in H9c2 cells

**Fig. 6:**
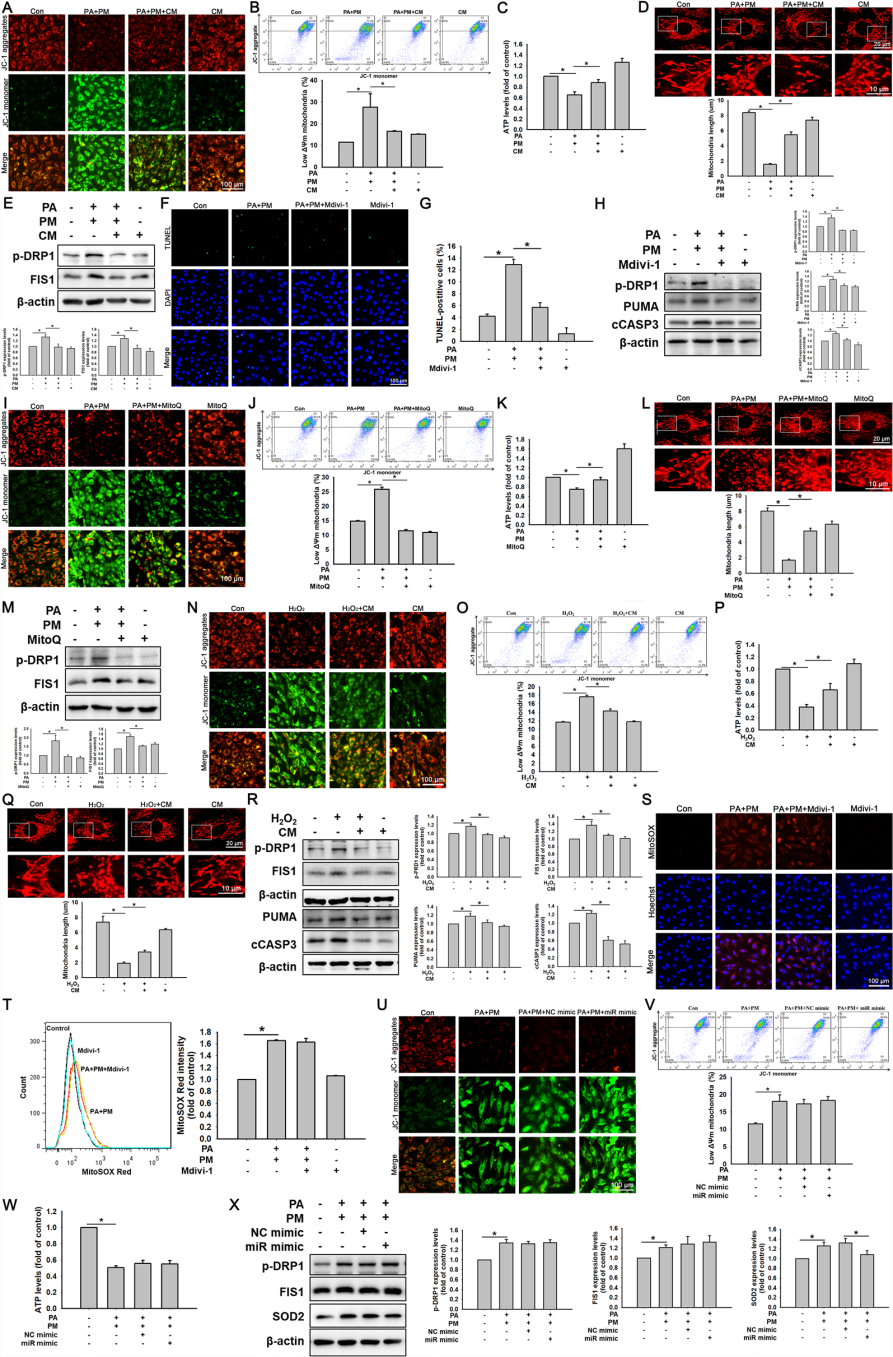
Effects of ADSC-CM on PA- and PM-induced mitochondrial fission in H9c2 cells and the relationship between mitochondrial fission and ROS production. **A**, **B** H9c2 cells were treated with 50 µM PA, 20 µg/mL PM, and CM for 24 h. ΔΨm was assessed by JC-1 staining via fluorescence microscopy (**A**; scale bar = 100 µm) and flow cytometry (**B**). **C** ATP assay evaluated mitochondrial function. **D** Mitochondrial morphology was examined using MitoTracker. Scale bars = 20 µm (upper), 10 µm (lower). **E** Western blot analyzed p-DRP1 and FIS1 expression. **F**, **G** TUNEL assay (F; green: TUNEL-positive; blue: nuclei) and quantification (**G**) assessed Mdivi-1 (10 µM) pretreatment effects on apoptosis. **H** Western blot examined p-DRP1, PUMA, and cCASP3 expression. **I**–**K** Effects of MitoQ on ΔΨm and ATP levels were assessed by JC-1 staining (**I**; scale bar = 100 µm), flow cytometry (**J**), and ATP assay (**K**). **L**, **M** MitoTracker staining and Western blot assessed the effects of MitoQ (100 nM) on mitochondrial morphology. Scale bars = 20 µm (upper), 10 µm (lower) (**L**) and protein expression (**M**) in H9c2 cells treated with PA and PM. **N**–**R** H9c2 cells were treated with H_2_O_2_ (100 µM) and CM for 24 h. The changes of ΔΨm (**N**, **O**), ATP levels (**P**), mitochondrial morphology (**Q**), and protein expression (**R**) were analyzed. **S**, **T** mROS levels were assessed by MitoSOX Red staining (**S**; scale bar = 100 µm) and flow cytometry (**T**) after Mdivi-1 and PA + PM treatment. **U**–**W** Cells transfected with negative control (NC) or *miR221/222* mimics for 24 h and then treated with PA and PM for 24 h. ΔΨm was examined by JC-1 (**U**; scale bar = 100 µm) and flow cytometry (**V**); ATP levels were measured (**W**). **X** Western blot assessed expression of p-DRP1, FIS1, and SOD2. The experiments were conducted at least five times, and the quantitative data were presented as mean ± SEM. *P < 0.05 is defined as statistically significant

To study the effect of ADSC-CM on mitochondrial function in H9c2 cells cotreated with PA and PM, the ΔΨm was examined via JC-1 staining. Control cells fluoresced red, had minimal green fluorescence, and displayed a normal ΔΨm (Fig. 6A). Cells treated with PA and PM for 24 h emitted green fluorescence, with slight red fluorescence, and presented a relatively low ΔΨm. However, ADSC-CM treatment significantly reduced these effects, and flow cytometry confirmed these results (Fig. 6B). Furthermore, compared with the Con group, the PA + PM group presented significantly lower ATP concentrations, whereas ADSC-CM increased ATP production (Fig. 6C).

To elucidate whether PA- and PM-induced apoptosis is related to mitochondrial fission, H9c2 cells were stained with MitoTracker. As shown in Fig. 6D, PA + PM treatment reduced the mitochondrial length compared to the control group. Conversely, ADSC-CM increased the mitochondrial length in H9c2 cells under PA + PM conditions. PA and PM increased the levels of the mitochondrial fission-associated proteins p-DRP1 and FIS1 expression. However, ADSC-CM reduced these effects (Fig. 6E; full-length blots are presented in Figure S11). Mdivi-1, a mitochondrial fission inhibitor, reduced PA- and PM-induced apoptosis and mitochondrial fission (Fig. 6F–H; full-length blots are presented in Figure S12). These results indicate that combined treatment with PA and PM induced apoptosis by increasing mitochondrial fission in H9c2 cells, whereas ADSC-CM attenuated this effect. To further confirm the correlation between ROS production and mitochondrial fission, PA- and PM-treated cardiomyocytes were incubated with MitoQ and subjected to fluorescent microscopy and flow cytometry via JC-1 staining (Fig. 6I and J). MitoQ treatment increased the low ΔΨm that was induced by PA and PM. MitoQ treatment also increased the ATP production and mitochondrial length in PA- and PM-treated H9c2 cells (Fig. 6K and L). As shown in Fig. 6M (full-length blots are provided in Figure S13), H9c2 cells treated with MitoQ under PA + PM conditions presented significantly reduced levels of p-DRP1 and FIS1. To further investigate the role of ROS in regulating mitochondrial fission, we used H₂O₂ (100 µM, 24 h) to elevate mROS levels. H₂O₂ treatment significantly reduced ΔΨm (Fig. 6N and O), reduced ATP levels (Fig. 6P), shortened mitochondrial length (Fig. 6Q), and upregulated p-DRP1 and FIS1 protein expression (Fig. 6R; full-length blots are presented in Figure S14), indicating that ROS promotes mitochondrial fission. In addition, we also detected whether Mdivi-1 could attenuate ROS production. As shown in Fig. 6S and T, Mdivi-1 treatment did not significantly alter PA + PM-induced mROS levels, indicating that ROS generation is not the downstream of mitochondrial fission in this model. On the basis of these results, loss- and gain-of-function analyses confirmed that ROS production activated mitochondrial fission and led to apoptosis in H9c2 cells. The relationships among ROS, mitochondrial fission, and *miR221/222* in H9c2 cells treated with PA and PM were further explored. H9c2 cells were transfected with *miR221/222* mimics and treated with PA and PM, followed by JC-1 staining and ATP assays. As shown in Fig. 6U–W, the *miR221/222* mimics failed to reverse the changes in the ΔΨm and ATP levels in PA + PM-treated H9c2 cells. Additionally, Western blot analysis showed the treatment with the *miR221/222* mimics did not reduce the levels of p-DRP1 and FIS1 but significantly reduced the expression of SOD2 (Fig. 6X; full-length blots are presented in Figure S15). These results strongly suggest that ADSC-CM inhibits the production of ROS, thereby reducing PA + PM-induced cardiomyocyte apoptosis. This pathway has no positive correlation with *miR221/222* levels.

## Discussion

Currently, obesity has become a global epidemic, and increasing evidence shows that HFD-induced obesity is a high-risk factor for impaired cardiac function. Additionally, epidemiological reports have shown that an increased risk of CVD is closely associated with long-term exposure to PM2.5 [48, 49]. Based on current environmental data, individuals living in areas with high ambient PM2.5 levels in developing countries may inhale up to 16 mg/kg PM2.5 annually, significantly when average concentrations exceed 200 μg/m^3^ [50–52]. Mice were intratracheally instilled with PM at 10 mg/kg once a week for two weeks, resulting in a cumulative exposure of 20 mg/kg. We employed the mouse model reflects a subacute high-dose scenario, approximately equivalent to 1–2 months of realistic exposure in a highly polluted urban environment. It has been reported that air pollution can exacerbate cardiovascular diseases caused by obesity [17]. This study revealed that an HFD and PM could reduce *miR221/222* levels, causing cardiac dysfunction and increasing the expression of apoptosis-related proteins. However, ADSC-CM treatment effectively alleviated HFD- and PM-induced cardiac dysfunction and cell apoptosis. ADSC-CM protected the heart from HFD- and PM-induced cardiomyocyte apoptosis via two pathways. One pathway is related to large amounts of *miR221/222* in ADSC-CM, which can reduce cardiac cell apoptosis by targeting PUMA and further reducing the activity of downstream apoptotic pathways. Another major pathway is related to the ability of ADSC-CM to reduce the production of ROS and thus reduce cardiomyocyte apoptosis.

ADSCs have been widely used in various clinical fields in recent years, including the treatment of chronic wounds [53], multiple sclerosis [54], Crohn’s disease [55], chronic discogenic low back pain [56], stress urinary incontinence [57], and myocardial ischemia [23]. ADSCs are easy to obtain by liposuction and are relatively free of ethical issues compared with other sources of stem cells. Furthermore, ADSCs have low senescence levels, are nonimmunogenic, and have multipotent differentiation potential, which makes them superior to stem cells from other sources [58]. Our previous study revealed that ADSC-CM could reduce cardiac apoptosis during I/R injury through *miR221/222* [23]. Our present study demonstrated that cardiomyocytes could take up and internalize ADSC-CM, which resulted in the inhibition of apoptosis by downregulating PUMA expression in PA + PM-treated H9c2 cells and HFD + PM-treated mice. Based on these results, ADSC-CM can be used to improve cardiomyocyte dysfunction therapeutically.

Abnormal levels of miRNA expression have been described in many reports associated with pathological events such as ischemia/reperfusion and heart failure [59]. *MiR221/222* are encoded in tandem on chromosome X with identical seed sequences. Our previous studies revealed that *miR221/222*-containing conditioned media [23] and exosomes [60] from adipose stem cells could protect against ischemia/reperfusion-induced cardiac injury. We also reported in earlier publications that *miR221/222* regulated TNF-α-induced ICAM-1 expression in endothelial cells [61] and lung epithelial cells [62]. Additionally, some studies have shown that *miR221/222* regulate the cell cycle by targeting p27 and p57 [63, 64]. Previous research has also demonstrated that targeting PUMA with *miR221/222* promotes glioblastoma cell survival [65]. Herein, we confirmed the high levels of *miR221/222* in ADSC-CM by qPCR and performed various studies on the cardioprotective mechanisms of *miR221/222* using cell culture and animal models. The data indicated that ADSC-CM contained more *miR221/222* than H9c2-CM did, which is consistent with our earlier experimental results. In addition, bioinformatics analysis revealed that the 3′ UTR of the PUMA mRNA contained a highly conserved predicted binding site for *miR221/222*. In this study, our data clearly revealed that PA + PM or HFD + PM conditions reduced the levels of *miR221/222*. Treatment of cells with ADSC-CM, which is enriched with *miR221/222*, or transfection with *miR221/222* mimics resulted in significant downregulation of PUMA expression. However, transfection with *miR221/222* inhibitors failed to reduce PUMA expression, suggesting that *miR221/222* in ADSC-CM may ameliorate PA + PM- or HFD + PM-induced cardiac injury by downregulating PUMA. We also used *miR221/222* KO and *miR221/222* TG mice to demonstrate that *miR221/222* could attenuate HFD + PM-induced cardiomyocyte apoptosis. We showed that *miR221/222* affected cell survival by targeting PUMA. The administration of large amounts of *miR221/222* to cardiomyocytes can protect the heart from damage caused by exposure to stimuli in the external environment.

Dysregulated ROS generation and oxidative stress are associated with CVDs, including cardiac hypertrophy, atherosclerosis, heart failure, ischemia/reperfusion injury, and diabetic cardiomyopathy [66–70]. Previous studies have shown that PA induces ROS accumulation, leading to cardiac cell apoptosis and inflammation [71, 72]. In addition, PM reduces cell viability, promotes cell apoptosis, and increases the intracellular ROS levels in H9c2 cells [73]. PM is involved in the pathogenesis of CVDs such as arrhythmia and hypertension by increasing the generation of ROS [74, 75]. This study revealed that PA and PM significantly induced excessive ROS production and increased H9c2 cell apoptosis. However, pretreatment with MitoQ (a ROS inhibitor) attenuated PA + PM-induced ROS generation and cell apoptosis. Notably, ADSC-CM effectively reduced PA + PM-induced ROS production. In addition, we found that ADSC-CM treatment increased the expression of SOD2, a well-known antioxidant enzyme. Increased SOD2 expression may be beneficial for reducing ROS production. During diabetic cardiomyopathy, SOD2 can protect cardiac morphology and ultimately normalize cardiomyocyte contractility [76]. Therefore, we believe that ADSC-CM inhibits ROS production by increasing SOD2 expression. However, cell transfection with the *miR221/222* inhibitor failed to reduce ROS generation. Studies have shown that *miR222-3p* directly binds to the 3′ UTR of SOD2. Furthermore, treating human cardiomyocytes with *miR222* mimics or inhibitors reduced or increased SOD2 expression, respectively [77]. These findings are consistent with our experimental results showing that *miR221/222* could not reduce PA + PM-induced ROS production. These results indicate that ADSC-CM attenuated PA + PM-induced apoptosis via the following two pathways: (1) abundant *miR221/222* in ADSC-CM reduced PUMA expression and (2) ADSC-CM reduced ROS production.

Accumulating evidence suggests that changes in mitochondrial morphology affect cell apoptosis and that a decreased ΔΨm is associated with mitochondrial dysfunction. A previous study revealed that when HL-1 cardiomyocytes were exposed to ischemia, mitochondria underwent fission and produced many fragments [78]. Regulating the mitochondrial membrane modification process through dynamic family proteins could reduce excessive mitochondrial fusion and fission in H9c2 cells after ischemia/reperfusion-like injury, improving the survival rate of cells, reducing ROS levels, and inhibiting cell apoptosis [79, 80]. In the present study, PA and PM reduced the ΔΨm and ATP production and increased the p-DRP1 and FIS1 expression levels, which are associated with mitochondrial fission. These findings are consistent with the previous report, which showed that PA exposure induces mitochondrial fission, characterized by the activation and phosphorylation of DRP1, a central regulator of mitochondrial dynamics [81]. Furthermore, exposure of offspring rats to PM2.5 was reported to increase the levels of DRP1 and FIS1, leading to cardiac developmental toxicity [82]. In contrast, the administration of ADSC-CM under PA and PM conditions reversed the ΔΨm change, increased ATP production, and inhibited the levels of p-DRP1 and FIS1. Furthermore, the inhibition of mitochondrial fission with the DRP1 inhibitor Mdivi-1 attenuated PA + PM-induced apoptosis. ROS are products of oxidative phosphorylation in mitochondria and play vital roles in cell signaling and developmental processes at low concentrations. However, when ROS are overproduced in defective mitochondria, they may act as cytotoxic factors, leading to excessive inflammation and apoptosis [83, 84]. A previous study revealed that ROS overproduction occurred during acute I/R injury, leading to mitochondrial fission [85]. In this study, we used MitoQ to clarify whether PA + PM-induced mitochondrial fission was due to excess ROS. The present findings showed that PA and PM could increase the production of ROS, thereby triggering mitochondrial fission and inducing the apoptosis of H9c2 cells. ADSC-CM treatment increased H9c2 cell survival, reduced ROS levels and mitochondrial fission, and prevented apoptosis.

## Conclusions

**Fig. 7:**
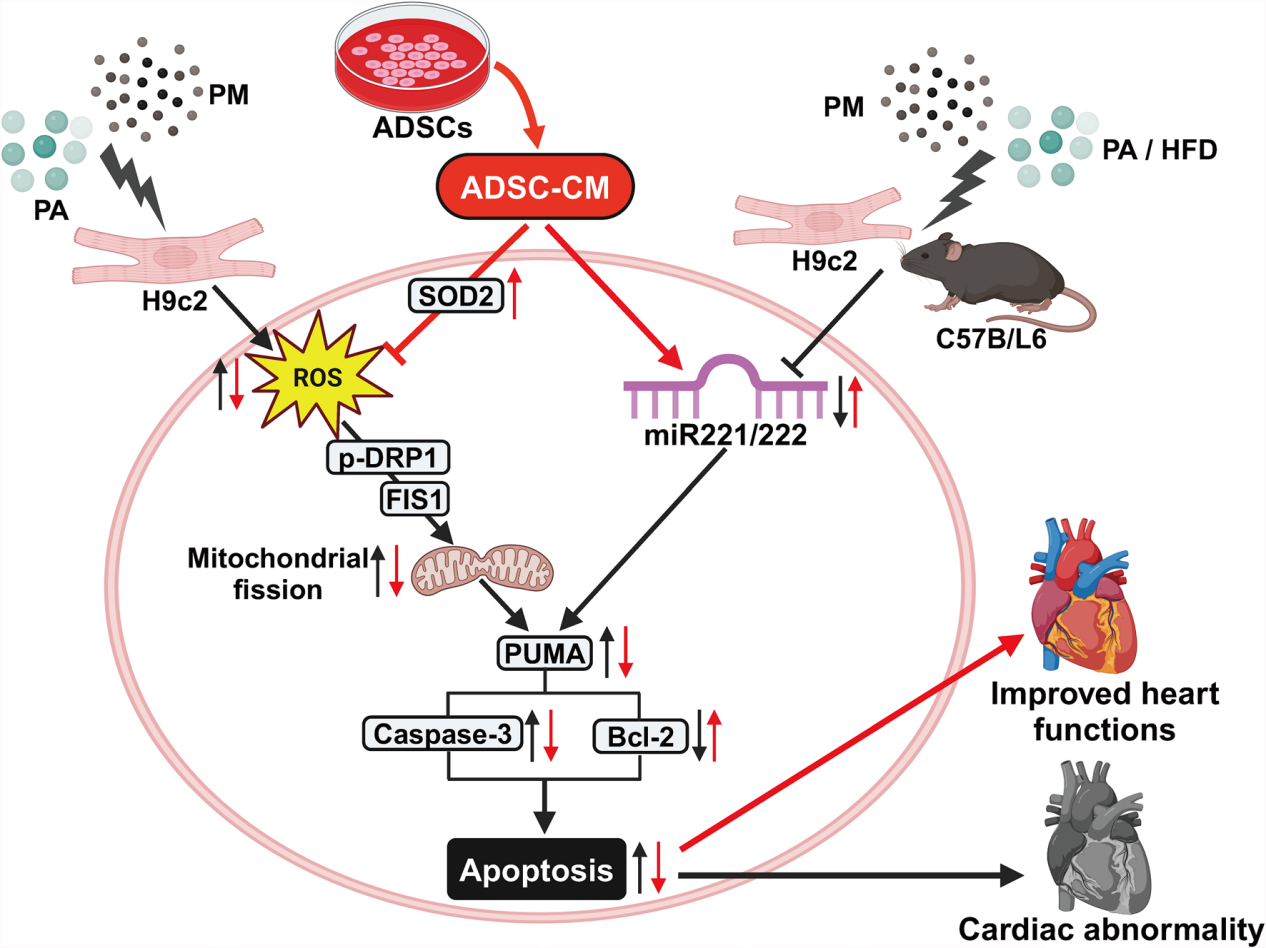
A graphical abstract summarizing the study findings. This schematic illustration summarizes the effects of obesity and air pollution on cardiomyocyte apoptosis and the potential protective role of ADSC-CM. In vitro, PA treatment and PM exposure simulated obesity and air pollution, respectively. In vivo, an HFD and PM were combined to replicate these conditions. This study revealed that ADSC-CM could reduce PA/HFD + PM-induced cardiomyocyte apoptosis in vitro and in vivo studies

Obesity increases the risk of cardiovascular disease, and environmental pollution exacerbates this risk. Overall, we found that PA and PM stimulation simultaneously suppressed *miR221/222* levels and increased ROS production, thereby inducing cardiomyocyte apoptosis. ADSC-CM reduced PA + PM-induced cardiomyocyte apoptosis by regulating *miR221/222* levels and inhibiting ROS production. Animal experiments confirmed that *miR221/222* in ADSC-CM could improve cardiac function and prevent damage (Fig. 7). ADSC-CM can serve as a therapeutic agent to alleviate PA + PM-induced apoptosis via two signaling pathways. However, the composition of ADSC-CM is complex, and its mechanism of reducing ROS generation is still unclear. Although this study focused on *miR221/222*, future studies should employ proteomic and transcriptomic analyses to characterize other bioactive components in ADSC-CM and determine their individual and synergistic effects. Therefore, the specific mechanism still needs further study. Furthermore, clinical translation must address several key challenges. The long-term efficacy and safety of ADSC-CM need to be further verified. By overcoming these mechanistic and translational challenges, ADSC-CMs could become promising therapeutic agents to alleviate obesity- and pollution-induced cardiac dysfunction.

## Supplementary Information

Additional file 1.

## Data Availability

All additional files are included in the manuscript.

## Abbreviations

ADSCs: Adipose-derived stem cells
ADSC-CM: Adipose-derived stem cell-conditioned medium
B6: C57B/L6 mice
cCASP3: Cleaved caspase-3
CVD: Cardiovascular disease
DMEM: Dulbecco’s modified Eagle’s medium
EF: Ejection fraction
FS: Fractional shortening
HFD: High-fat diet
IVCT: Left ventricular isovolumetric contraction time
IVRT: Left ventricular isovolumetric relaxation time
KO: *miR221/222* Knockout mice
LVPWd: Diastolic left ventricular posterior wall thickness
LVPWs: Systolic left ventricular posterior wall thickness
miR inhibitor: *miR221/222* Inhibitor
miR mimic: *miR221/222* Mimic
miRKD-CM: CM obtained after knockdown of *miR221/222* in ADSCs
NC inhibitor: Negative control inhibitor
NC mimic: Nnegative control mimic
NCKD-CM: CM obtained after knockdown negative control in ADSCs
PA: Palmitic acid
PM: Particulate matter
ROS: Reactive oxygen species
SV: Stroke volume
TG: *miR221/222* Transgenic mice
