# Supplementary material for: MiR221/222 in the conditioned medium of adipose-derived stem cells attenuates particulate matter and high-fat diet-induced cardiac apoptosis
Source: Stem Cell Res Ther. 2025 Jun 3;16:285. doi: 10.1186/s13287-025-04381-8 (PMC12135233; doi:10.1186/s13287-025-04381-8)
Supplement: Supplementary file 1 — Additional file 1. [file 13287_2025_4381_MOESM1_ESM.docx]

**Full-length blots**

**Figure S1. Full-length blots of western blot images in Fig. 1C.**


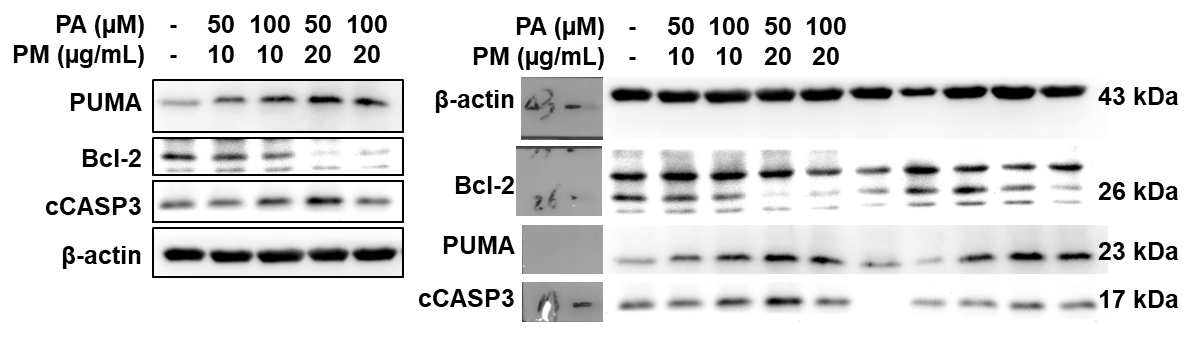


**Figure S2. Full-length blots of western blot images in Fig. 1E.**

(A: β-actin, PUMA, cCASP3; B: β-actin, Bcl-2)
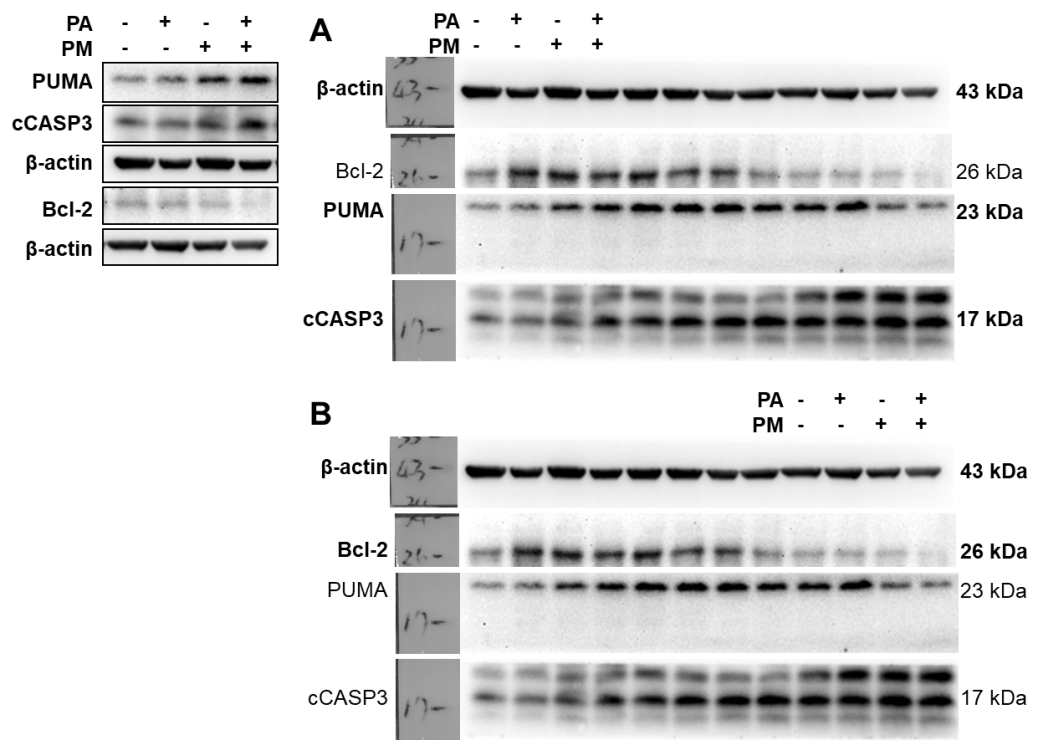


**Figure S3. Full-length blots of western blot images in Fig. 1J.
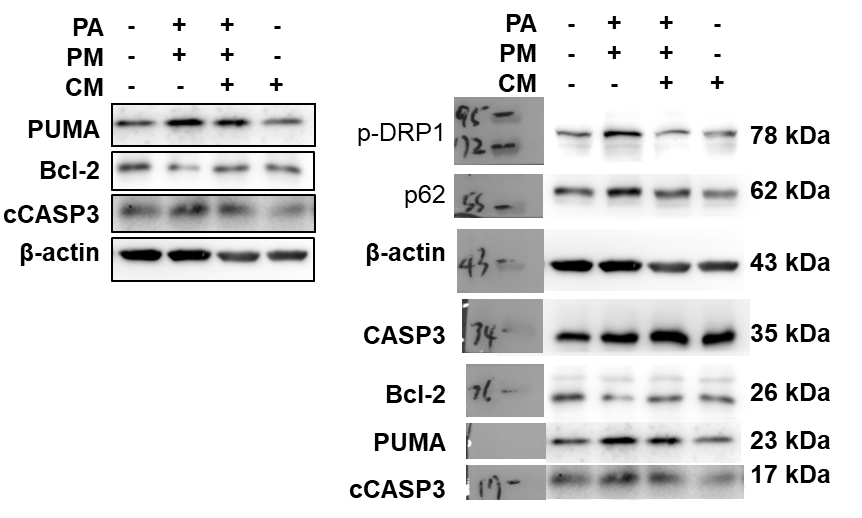
**

**Figure S4. Full-length blots of western blot images in Fig. 2D.**

(A: β-actin, PUMA, cCASP3; B: β-actin, Bcl-2)**
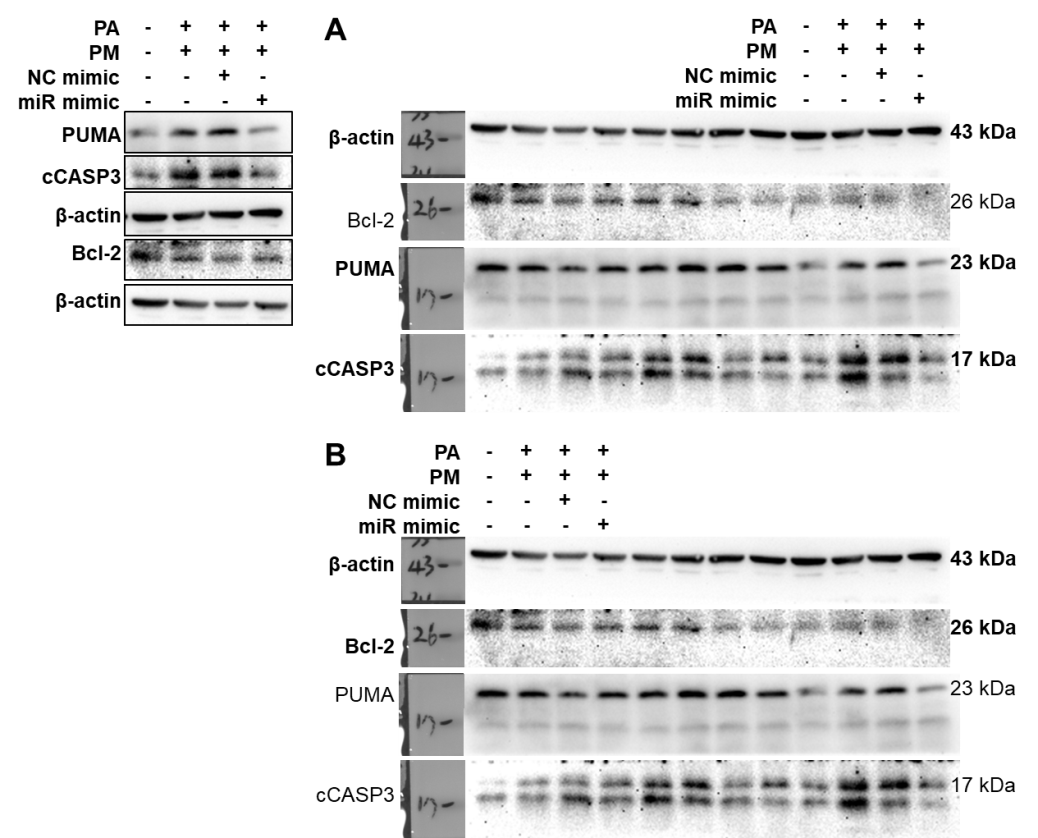
**

**Figure S5. Full-length blots of western blot images in Fig. 2F.
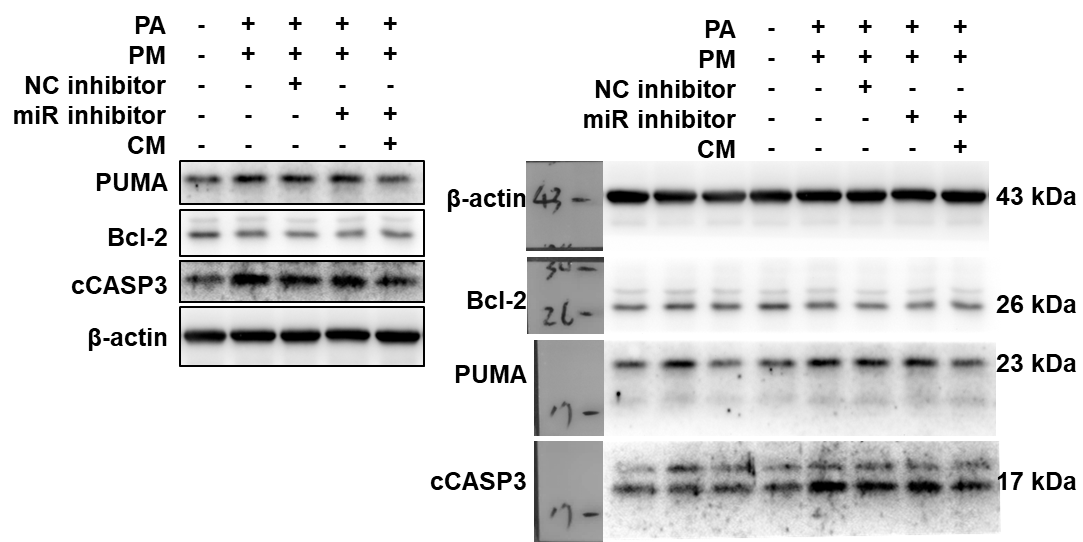
**

**Figure S6. Full-length blots of western blot images in Fig. 2J.**

(A: β-actin, PUMA, cCASP3; B: β-actin, Bcl-2)**
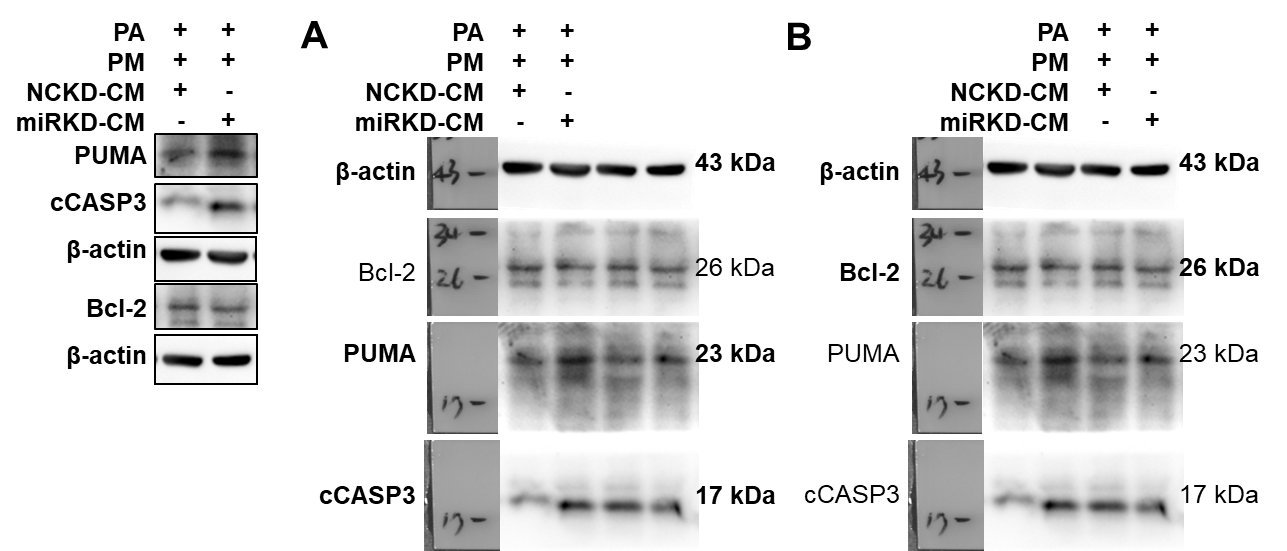
**

**Figure S7. Full-length blots of western blot images in Fig. 3Q.
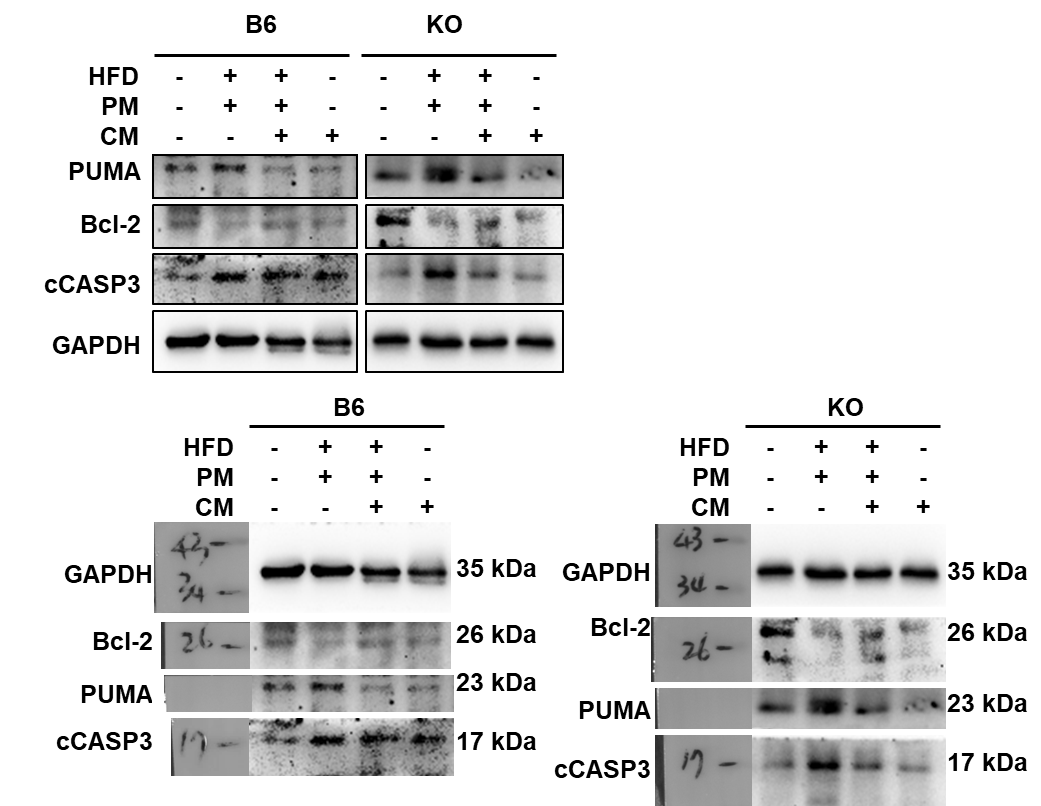
**

**Figure S8. Full-length blots of western blot images in Fig. 4P.
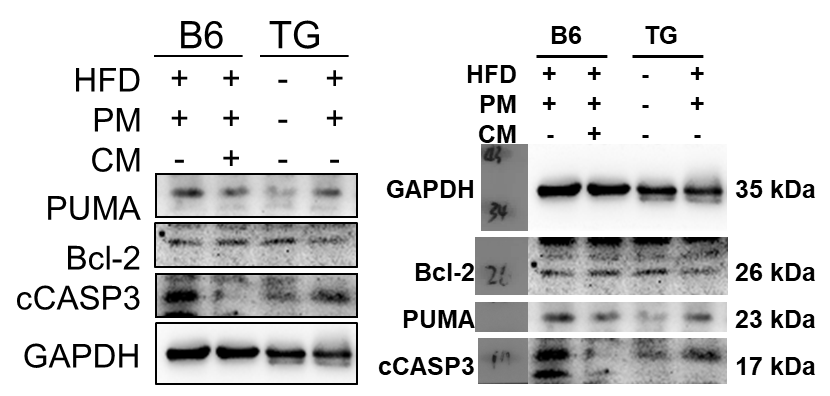
**

**Figure S9. Full-length blots of western blot images in Fig. 5E.
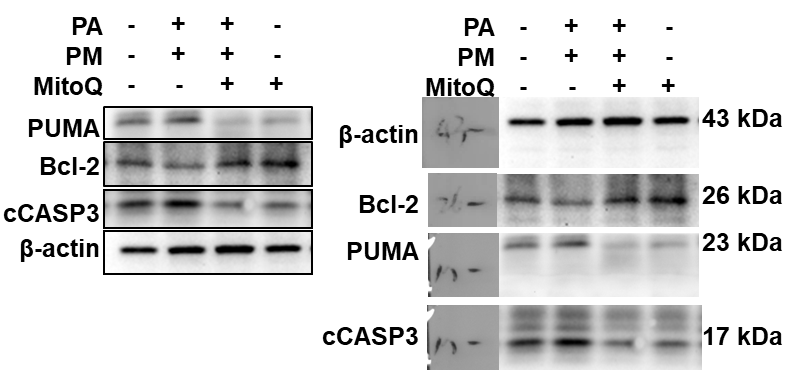
**

**Figure S10. Full-length blots of western blot images in Fig. 5J.**

(A: β-actin, SOD2, SOD1; B: SOD3)**
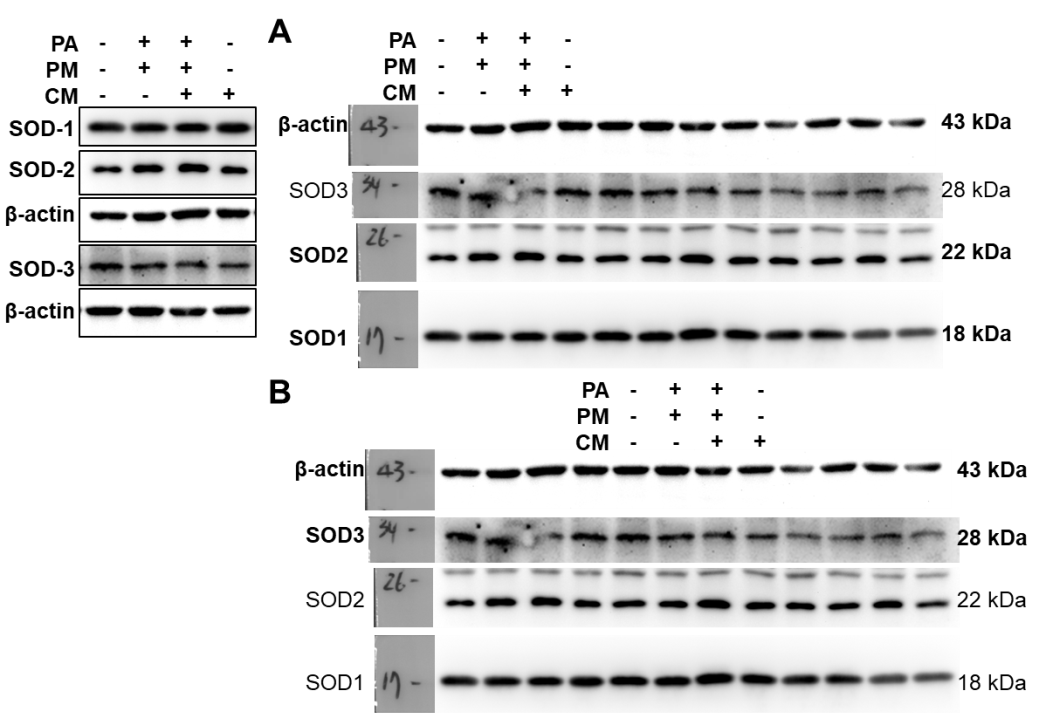
**

**Figure S11. Full-length blots of western blot images in Fig. 6E.
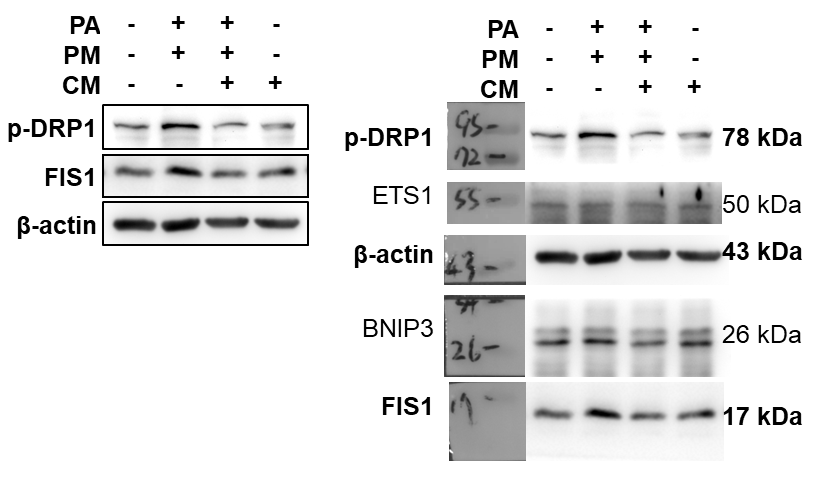
**

**Figure S12. Full-length blots of western blot images in Fig. 6H.
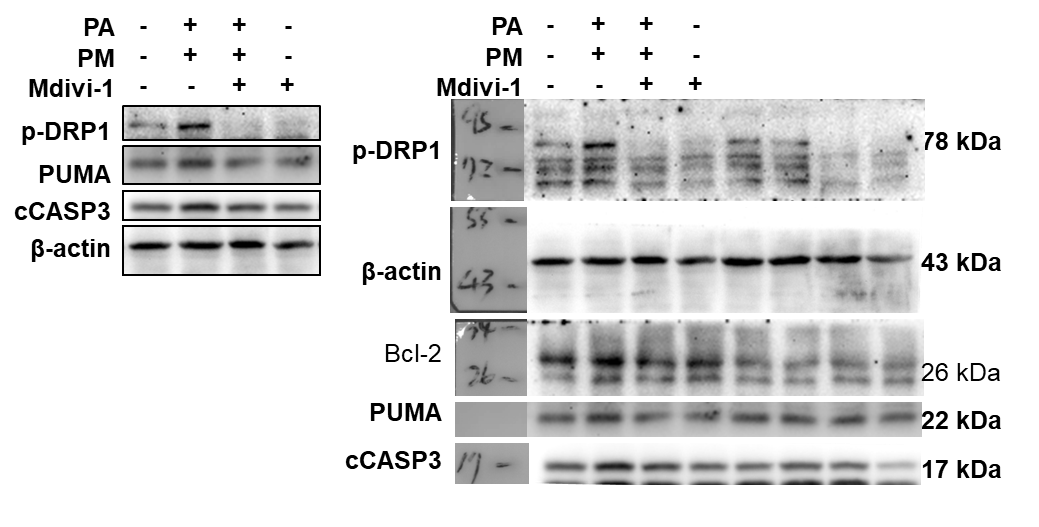
**

**Figure S13. Full-length blots of western blot images in Fig. 6M.**

**
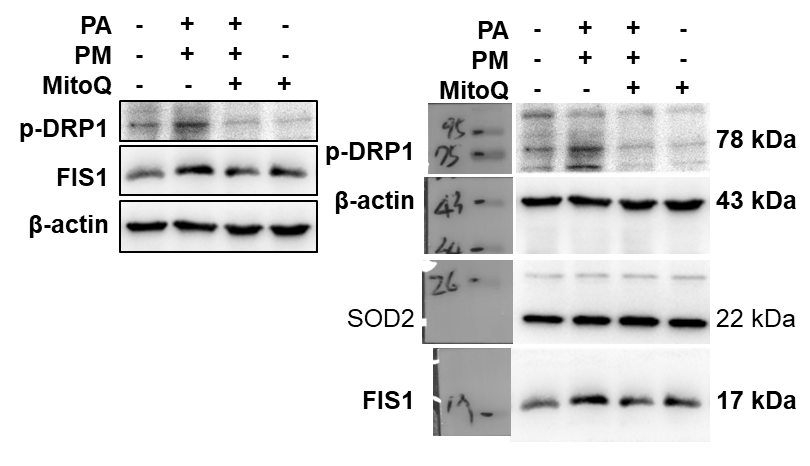
**

**Figure S14. Full-length blots of western blot images in Fig. 6R.**

(A: p-DRP1, β-actin, FIS1; B: β-actin, PUMA, cCASP3)

**
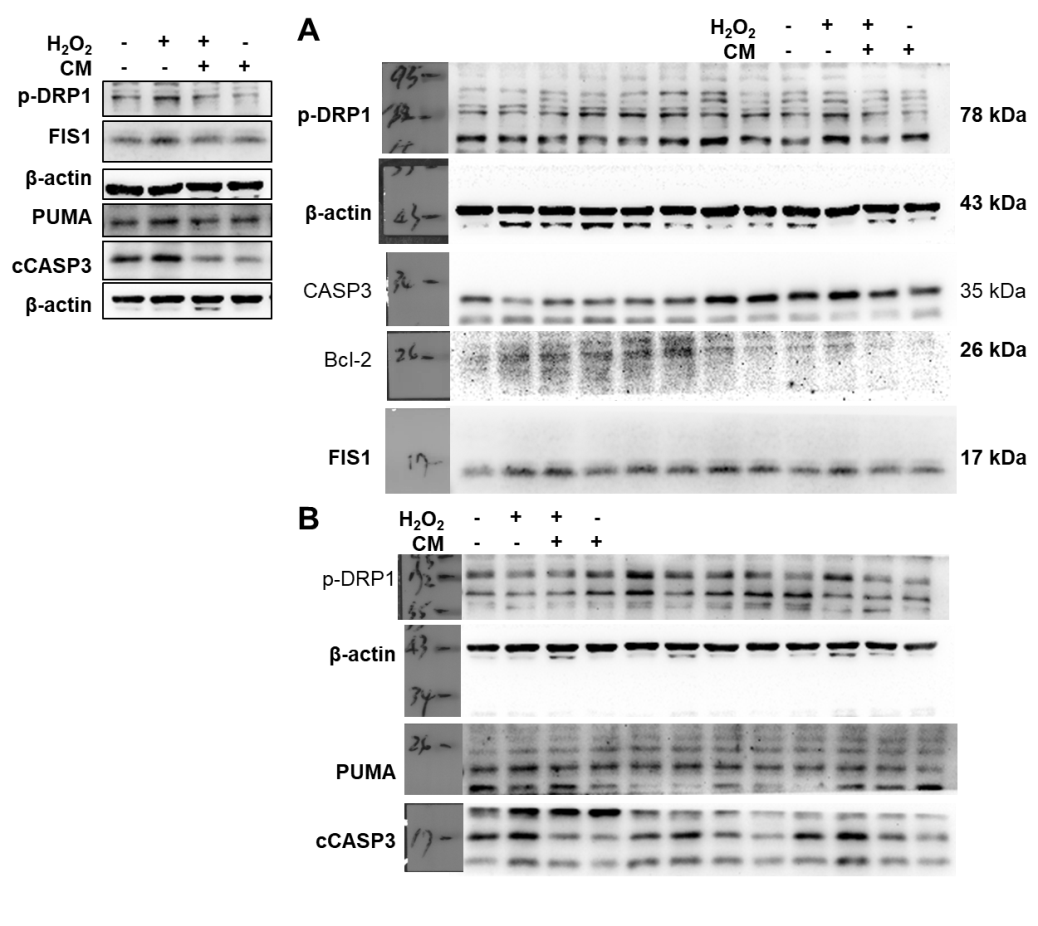
**

**Figure S15. Full-length blots of western blot images in Fig. 6X.
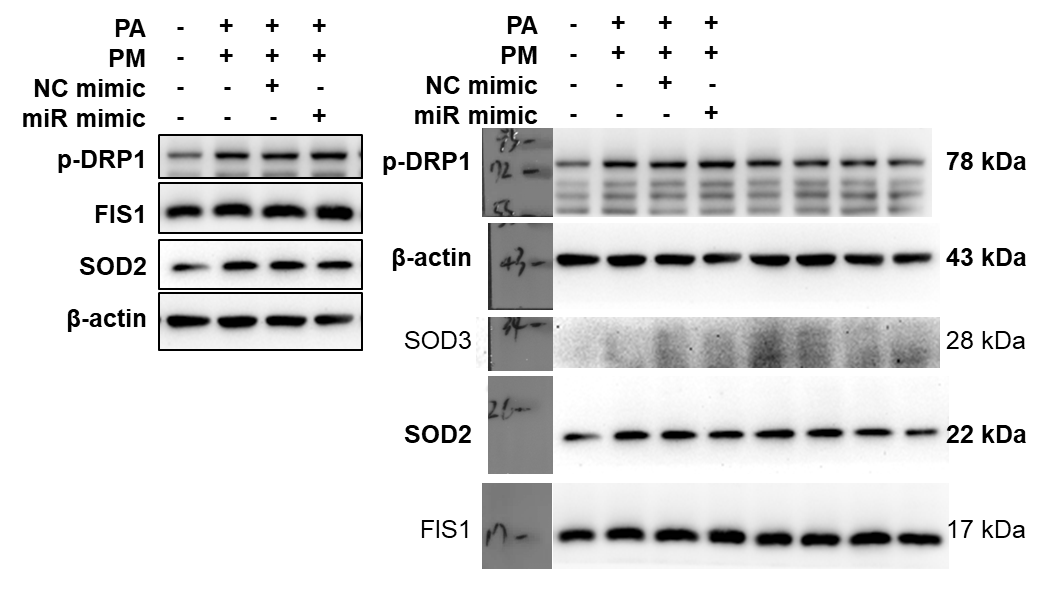
**
